# Niche- and Gender-Dependent Immune Reactions in Relation to the Microbiota Profile in Pediatric Patients with Otitis Media with Effusion

**DOI:** 10.1128/IAI.00147-20

**Published:** 2020-09-18

**Authors:** Frida Enoksson, Alicia Ruiz Rodriguez, Chikondi Peno, Carlos Balcazar Lopez, Fredrik Tjernström, Debby Bogaert, Anders P. Hakansson, Caroline Bergenfelz

**Affiliations:** a Department of Otorhinolaryngology, Helsingborg Hospital, Helsingborg, Sweden; b Centre for Inflammation Research, Queen’s Medical Institute, University of Edinburgh, Edinburgh, United Kingdom; c Department of Otorhinolaryngology, Skåne University Hospital, Lund, Sweden; d Department of Translational Medicine, Experimental Infection Medicine, Lund University, Malmö, Sweden; University of California, Davis

**Keywords:** cytokines, immune cells, inflammatory mediators, microbiota, mucosal pathogens, nasopharynx, otitis media, otopathogens, respiratory tract

## Abstract

Otitis media with effusion (OME) is a common inflammatory disease that primarily affects children. OME is defined as a chronic low-grade inflammation of the middle ear (ME), without any signs of infection and with effusion persisting in the ME for more than 3 months. The precise pathogenesis is, however, not fully understood. Here, we comprehensively characterized and compared the host immune responses (inflammatory cells and mediators) and the overall microbial community composition (microbiota) present in matched middle ear effusion (MEE) samples, external ear canal (EEC) lavages, and nasopharynx (NPH) samples from children with OME. Female patients had significantly increased percentages of T lymphocytes and higher levels of a wide array of inflammatory mediators in their MEE compared to that of male patients, which were unrelated to microbiota composition. The relative abundances of identified microorganisms were strongly associated with their niche of origin. Furthermore, specific inflammatory mediators were highly correlated with certain bacterial species. Interestingly, some organisms displayed a niche-driven inflammation pattern in which presence of Haemophilus spp. and Corynebacterium propinquum in MEE was accompanied by proinflammatory mediators, whereas their presence in NPH was accompanied by anti-inflammatory mediators. For Turicella and Alloiococcus, we found exactly the opposite results, i.e., an anti-inflammatory profile when present in MEE, whereas their presence in the the NPH was accompanied by a proinflammatory profile. Together, our results indicate that immune responses in children with OME are highly niche- and microbiota-driven, but gender-based differences were also observed, providing novel insight into potential pathogenic mechanisms behind OME.

## Abstract (precis)

Otitis media with effusion (OME) is a common inflammatory disease that primarily affects children. OME is defined as a chronic low-grade inflammation of the middle ear (ME), without any signs of infection and with effusion persisting in the ME for more than 3 months. The precise pathogenesis is, however, not fully understood. Here, we comprehensively characterized and compared the host immune responses (inflammatory cells and mediators) and the overall microbial community composition (microbiota) present in matched middle ear effusion (MEE) samples, external ear canal (EEC) lavages, and nasopharynx (NPH) samples from children with OME.

## INTRODUCTION

Otitis media (OM) is a common inflammatory condition of the middle ear that predominantly affects children. With more than 700 million cases annually, OM accounts for a majority of pediatric emergency department visits and antibiotic prescriptions worldwide (1). OM presents itself as a continuum of symptoms with acute or chronic characteristics. Acute otitis media (AOM) is associated with an abrupt onset, considerable pain, and clear signs of infection and inflammation in the middle ear (ME). In contrast, otitis media with effusion (OME), is defined as a chronic and low-grade inflammation of the ME, without signs of acute infection (e.g., no fever or earache) and with nonpurulent effusion persisting in the ME for more than 3 months (2, 3). This prolonged accumulation of effusion causes a conductive hearing loss, which makes OME the most common cause of reversible hearing loss in children. If untreated, it may over time lead to speech delay, as well as cognitive and developmental problems (4, 5). OME can occur as a sequela of AOM, but the precise pathogenesis is still not fully understood, including the mechanisms contributing to initiation, continuation, and spontaneous resolution of inflammation, as well as the relative roles and the interplay of the microbiota (pathogens and normal flora), the host immune response, and other host characteristics.

With the advancement of culture-independent techniques such as 16S rRNA-based sequencing, the complex landscape of the respiratory tract microbiota is starting to emerge. In patients with an intact tympanic membrane, OM is widely presumed to result from ascension of bacteria from the nasopharynx (NPH) through the Eustachian tube to the ME. This route is supported by the observation that colonization of specific bacteria in the NPH is associated with increased risk of OM, and is possibly even a prerequisite for particular infections (6, 7). A strong correlation between the microbiota in the NPH and that in middle ear effusion (MEE) in patients with AOM with tympanostomy tubes has also been shown, further supporting this notion (8). The same study revealed high abundances of Pseudomonas aeruginosa, Staphylococcus aureus, Turicella otitidis, Klebsiella pneumoniae, and Haemophilus spp. in MEE, as well as in the NPH, in children with AOM (8). Remarkable similarities have also been described for the ME microbiota in recurrent AOM and OME (9). A microbiota profile dominated by the genera *Haemophilus*, Moraxella, and Streptococcus, as well as Alloiococcus otitidis, Turicella otitidis, and Corynebacterium spp. has been reported in the MEE from indigenous Australian children with chronic suppurative otitis media (CSOM), a patient group highly prone to develop chronic purulent OM (10, 11), but is also seen in MEE samples from other populations (12, 13). However, whether some of these organisms are indeed true pathogens, whether they are opportunistic organisms residing either in the NPH or in the external ear canal (EEC) that gain access to the ME subsequent to infection with classic otopathogens, or even whether they constitute contamination from the external ear canal, is still debated (14, 15).

Inflammation is a hallmark of OM and may be initiated when bacteria are recognized by the host. Thus, initiation of inflammation may be caused by infection, yet the role of microbes in perpetuating the inflammation in OME is debated (16). The specific immune cell populations present are, however, still not well characterized in MEE from patients with OME. Early reports suggested that macrophages are the most common immune cell population in all MEE regardless of appearance, with the exception of purulent MEE, such as in AOM, where neutrophils predominate (17, 18). Presence of T lymphocytes and B lymphocytes have also been reported to various extent in MEEs of serous or mucoid character (17, 19). Many studies have compared the composition of inflammatory mediators in serous and mucoid MEEs, with mucoid MEEs being shown to contain more inflammatory mediators such as interleukin-8 (IL-8) and RANTES (20). Tumor necrosis factor alpha (TNF-α), IL-1β, and IL-8 are commonly identified in MEE samples from patients with chronic OM, and otitis-prone children have lower expression of IL-6 and IL-8 in their MEEs (4, 21–23). Thus, a dysregulated or reduced innate inflammatory response may be related to disease progression in otitis-prone children, i.e., children prone to purulent AOM (21). The contribution of specific inflammatory cells and mediators in OME patients without a history of ear infections is less studied. It is not clear whether and how specific immune cell populations and factors are related to specific microorganisms in the same niche, nor is it clear how they contribute to the pathogenesis of polymicrobial OM. Furthermore, the exact composition of MEE and the nature of the inflammatory mediators and immune cells present likely varies depending on genetic and environmental factors, as well as on the specific microorganisms present.

The current literature is confounded by unclear definitions of OM/OME and is most often based on pooled samples from both ears, although the two ears possibly could differ in microbiota composition and active immunological processes at play (24). Moreover, few studies have characterized inflammatory mediators and cells in combination with the microbiota from the same sample. In this study, we aimed to comprehensively characterize, correlate, and compare the immune cells and inflammatory mediators with the microbiota present in matched MEE and NPH samples from patients with OME.

## RESULTS

### Clinical characteristics of the included patients.

Out of 53 patients eligible for the study, 36 patients were enrolled, and middle ear effusions (MEEs), external ear canal (EEC) lavage and nasopharynx (NPH) samples were collected when possible. Out of the 36 patients, nine had bilateral dry ears at the time of surgery and were hence excluded from all analyses (see Fig. S1 in the supplemental material for overview of inclusion and sample collection). The median age of the remaining 27 patients was 58 months (± standard error of the mean [SEM], 4.1 months). In total, 14 patients (51.9%) were male and 13 (48.1%) were female (see Table S1 in the supplemental material). There was no significant difference between the age at myringotomy for boys and girls. Clinical microbiological analyses identified microorganisms in 31.5% of the MEE samples analyzed and revealed Haemophilus influenzae as the most commonly identified species (*n *= 7; 13.0%), followed by Staphylococcus aureus (*n *= 4; 7.4%), coagulase-negative staphylococci (*n *= 4; 7.4%), *Alloiococcus otitidis* (*n *= 4; 7.4%), Moraxella catarrhalis (*n *= 1; 1.9%), Streptococcus pyogenes (*n *= 1; 1.9%), and Staphylococcus spp. (*n *= 1; 1.9%) (Table S1). In NPH samples, M. catarrhalis (*n *= 18; 66.7%) and H. influenzae (*n *= 9; 33.3%) predominated, followed by Streptococcus pneumoniae (*n *= 4; 14.8%), Streptococcus pyogenes (*n *= 3; 11.1%), S. aureus (*n *= 1; 3.7%), and Neisseria spp. (*n *= 1; 3.7%) comprising in total 88.9% of all NPH samples analyzed (Table S1).

### Microbiota profiles of OME samples: similarities between MEE and EEC.

**FIG 1:**
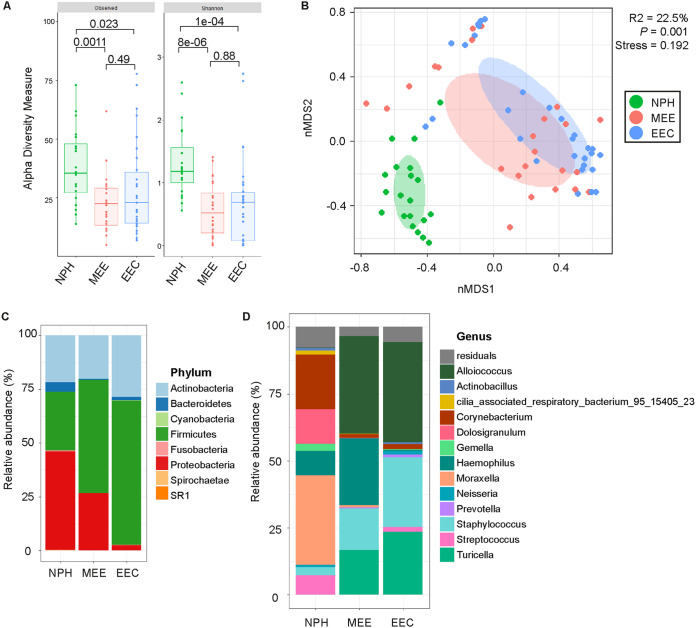
Total microbiota composition differs between NPH, MEE, and EEC lavage samples. (A) Box plots of the α-diversity indices of the microbiota across the three niches. Medians and interquartile ranges are shown. The significance of differences was calculated using the Mann-Whitney U test with Benjamini-Hochberg (BH) correction for multiple comparisons. Observed species and the Shannon diversity index were significantly higher within NPH samples (green) compared to those for MEE (red) and EEC lavage (blue) samples. (B) Nonmetric multidimensional scaling (nMDS) plot based on Bray-Curtis (BC) dissimilarity between samples, with data points and ellipses colored by niche as follows: NPH (green), MEEs (red), and EEC lavage (blue) samples, with NPH samples being significantly different in composition compared to MEE and EEC lavage samples, as determined by permutational analysis of variance (PERMANOVA) analysis based on Bray-Curtis dissimilarity index ordination. Ellipses represent the standard deviation of all points within a niche. (C and D) The mean relative abundance of indicated phyla (C) and genera (D) in NPH, MEE, and EEC lavage samples.

First, the microbiota composition was characterized by 16S rRNA-based sequencing from the matched MEE, NPH, and EEC lavage samples. Sufficient DNA for sequencing was obtained from 44% of MEE samples, 79% of EEC lavage samples, and 74% of NPH samples. After quality control and filtering out environmental contaminating operational taxonomic units (OTUs), as identified by the frequency method from the *decontam* R package, a total of 5,938,330 reads remained for analysis (mean ± SEM, 80,247.7 ± 6,722.04 reads per sample). These reads were binned into 140 OTUs, representing 73 taxonomic genera from 8 phyla. The observed number of species and the Shannon diversity index were significantly higher within NPH samples compared to those in MEE or EEC lavage samples (Fig. 1A). Furthermore, the total microbiota composition was significantly different between NPH and MEE samples as well as EEC lavage samples (β-diversity, *R*^2^ = 0.218, *P = *0.001) (Fig. 1B; see also Table S2 in the supplemental material). In MEE and EEC lavage samples, the local community was strongly dominated by Firmicutes taxa, which accounted for 63.5% and 54.6% of the total microbiota, respectively, with Alloiococcus and Staphylococcus being the predominant genera (Fig. 1C and D). In contrast, the predominant phylum in NPH samples was Proteobacteria, accounting for 46.1%, followed by *Firmicutes* and Actinobacteria with 27.5% and 21.6% of the relative abundance, respectively. *Proteobacteria* was represented by Moraxella and Haemophilus, *Firmicutes* by Dolosigranulum and Streptococcus, and *Actinobacteria* by Corynebacterium (Fig. 1C and D).

Hierarchical clustering at the OTU level showed that NPH samples had a distinct microbiota profile, largely separated from those of MEE and EEC lavage samples, and that was characterized by high microbial diversity dominated by *Moraxella* (5), Corynebacterium propinquum (6), *Streptococcus* (10), *Haemophilus* (4), and *Dolosigranulum* (9) (see Fig. S2 in the supplemental material). MEE and EEC lavage samples had overall similar microbiota profiles that were dominated by one or two of the following taxa: *Alloiococcus* (1), Staphylococcus epidermidis (2), Turicella (3) and *Haemophilus* (4) (Fig. S2).

### Biomarker species in MEE and NPH samples.

**FIG 2:**
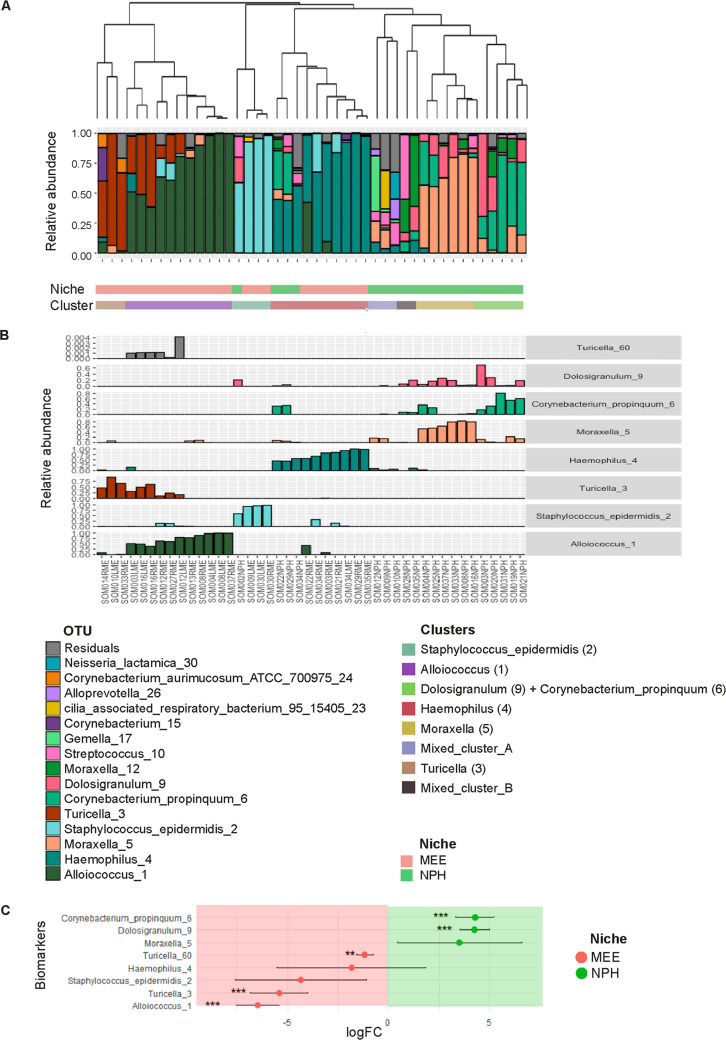
Identification of biomarker species in MEE and NPH samples. (A) Dendrogram visualizing an average linkage hierarchical clustering of MEE and NPH samples on the basis of the Bray-Curtis dissimilarity matrix. Stacked bar charts show the relative abundances of the 15 highest-ranked operational taxonomic units (OTUs) and of residual bacteria. Hierarchical clustering of MEE and NPH samples identified eight clusters. (B) Biomarker species for these eight profiles were determined using random forest analysis (*VSURF*). Classifier taxa for these eight clusters were as follows: cluster 1, S. epidermidis (2); cluster 2, *Alloiococcus* (1); cluster 3, *Dolosigranulum* (9) and *C. propinquum* (6); cluster 4, *Haemophilus* (4); cluster 5, *Moraxella* (5); cluster 6, mixed cluster with *Moraxella* (5) and *Haemophilus* (4); cluster 7, *Turicella* (3); and cluster 8, mixed cluster with *Dolosigranulum* (9), *C. propinquum* (6), and *Haemophilus* (4). See Fig. S4 in the supplemental material for significant differences in biomarkers abundance among clusters. (C) Associations between the biomarker species abundance and either the NPH niche or the MEE niche (determined by *metagenomeSeq*). *C. propinquum* (6) and *Dolosigranulum* (9) were significantly associated with NPH; *Alloiococcus* (1), *Turicella* (3), and *Turicella* (60) were significantly associated with the MEE samples. *, *P* < 0.05; **, *P* < 0.01; ***, *P* < 0.001.

Hierarchical clustering showed the presence of eight distinct microbiota profiles, which were mainly driven by the abundance of eight biomarker species (Fig. 2A and B). Most biomarker species were differentially abundant in NPH and MEE samples, except for Staphylococcus epidermidis (2), *Haemophilus* (4), and *Moraxella* (5), which were consistently present between niches (Fig. 2C). *Haemophilus* (4) was present in both niches, although with a higher, albeit nonsignificant, abundance in MEE samples. In contrast, Corynebacterium propinquum (6) and *Dolosigranulum* (9) were significantly more abundant in NPH samples, whereas *Alloiococcus* (1), *Turicella* (3), and *Turicella* (60) abundances were associated with MEE samples (Fig. 2C). To assess the relationship between clusters and biomarkers, we performed linear models with the log-transformed relative abundance of the biomarkers and the cluster identity, accounting for niche. We found significant associations for most of them; cluster 1 was associated with Staphylococcus epidermidis (2), cluster 2 with *Alloiococcus* (1), cluster 3 with Corynebacterium propinquum (6) and *Dolosigranulum* (9), cluster 4 with *Haemophilus* (4), cluster 5 with *Moraxella* (5), and cluster 7 with *Turicella* (3). In contrast, clusters 6 and 8 were not strongly associated with any biomarker species and are referred to here as Mixed_cluster_A and Mixed_cluster_B, respectively (see Fig. S3 in the supplemental material).

### General differences in the inflammatory profile of MEE and NPH samples.

Next, we assessed the overall inflammatory profiles (inflammatory cells and mediators) in MEE and NPH samples. MEE samples had nonsignificantly higher percentages of neutrophils (CD66b-positive [CD66b^+^] cells) and IL-6 concentrations than those in NPH samples (see Table S3 in the supplemental material). Conversely, NPH samples displayed significantly higher percentages of cytotoxic T lymphocytes (CD8^+^ cells) and B lymphocytes (CD19^+^ cells), as well as higher concentrations of the proinflammatory mediators TNF-α and IL-1β than those in MEE samples (Table S3). Memory T lymphocytes predominated over naive lymphocytes in all samples, although the percentages of naive CD8^+^ cytotoxic T lymphocytes were higher in MEE samples than in NPH samples (Table S3).

### The inflammatory profile in MEEs is correlated with gender but not with age.

As an age of <5 years and male gender are reported risk factors for OM (2), we stratified the MEE and NPH samples according to age (the median age of included patients, 58 months, was used as a cutoff) or by gender and assessed the inflammatory cells and mediators present in the samples. A significantly higher percentage of neutrophils (CD66b^+^ cells) was observed specifically in MEE samples, in children younger than 58 months compared with children older than 58 months (see Table S4 in the supplemental material). However, no other leukocyte population and inflammatory mediator investigated differed significantly between younger (<58 months) and older (>58 months) children in either MEE or NPH samples (Table S4).

**FIG 3:**
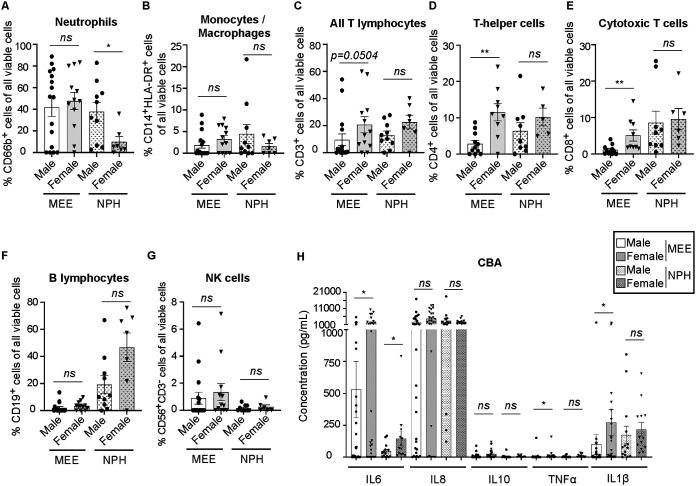
MEE samples from female patients display more T lymphocytes and inflammatory mediators. (A to G) Cells from MEE and NPH samples were analyzed by flow cytometry. All analyses were performed gated on viable (7-AAD^−^) cells and stratified based on sex (male, black circles and open bars; female, black triangles and gray bars). NPH samples are indicated in patterned bars. Percentages (%) of cells from individual samples (nondetectable subsets set to 0%), with mean and standard error of the mean (SEM) shown. Male MEE, *n *= 16; female MEE, *n *= 12; male NPH, *n *= 10; and female NPH, *n *= 7, unless otherwise stated. (A) Percentage (%) of CD66b^+^ granulocytes (predominantly neutrophils). (B) Percentage (%) of CD14^+^ HLA-DR^+^ monocytes/macrophages. (C) Percentage (%) of CD3^+^ T lymphocytes. (D) Percentage (%) of CD4^+^ T helper lymphocytes. Male MEE, *n *= 11; female MEE, *n *= 9; male NPH, *n *= 9; and female NPH, *n *= 5. (E) Percentage (%) of CD8^+^ cytotoxic T lymphocytes. Male MEE, *n *= 11; female MEE, *n *= 9; male NPH, *n *= 9; female NPH, *n *= 6. (F) Percentage (%) of CD19^+^ B lymphocytes. (G) Percentage (%) of CD56^+^ CD3^−^ natural killer (NK) cells. Male MEE, *n *= 16; female MEE, *n *= 12; male NPH, *n *= 10; female NPH, *n *= 6. (H) MEE and NPH samples were analyzed by cytokine bead array (CBA) for indicated inflammatory mediators. Concentration (pg/ml) in individual samples with mean and SEM shown. Male MEE, *n *= 28; female MEE, *n *= 26; male NPH, *n *= 14; female NPH, *n *= 13. All statistics by Mann-Whitney U test. *, *P* < 0.05; **, *P* < 0.01.

Interestingly, when analyzing the NPH and MEE samples in male and female patients, striking differences were apparent in the composition of both inflammatory cells and mediators, specifically in the MEEs (Fig. 3). NPH samples were overall similar in male and female patients, with the exception of a significant enrichment of CD66b^+^ neutrophils in male patients (Fig. 3A) and significantly higher concentrations of IL-6 in female patients (Fig. 3H). When investigating MEE samples, MEEs from female patients had a significantly higher proportion of CD4^+^ T helper and CD8^+^ cytotoxic T lymphocytes (Fig. 3D and E) and also displayed higher concentrations of the proinflammatory mediators IL-6, TNF-α, and IL-1β than those found in MEEs from male patients (Fig. 3H). In fact, most inflammatory mediators investigated were present at significantly higher concentrations in MEEs from female patients compared with those from male patients (Fig. S4). MCP-1 was the only factor that was higher in MEEs from male patients than in female patients, albeit not significantly so (Fig. S4).

### Innate immune cells are positively associated with a vast variety of inflammatory mediators, whereas adaptive immune cells correlate with specific mediators.

Inflammatory mediators (including cytokines and chemokines) are crucial players in initiating and maintaining inflammation and can be produced by epithelial cells, as well as by resident or recruited immune cells. When analyzing MEE samples using nonparametric Spearman’s correlation, the proportion of neutrophils (CD66b^+^ cells) and monocytes/macrophages (CD14^+^ HLA-DR^+^ cells) in MEE samples significantly correlated with the levels of a wide range of proinflammatory mediators, including IL-8, IL-1β, IL-6, TNF-α, and IL-12, but also with anti-inflammatory and angiogenesis-inducing factors such as IL-10, basic fibroblast growth factor (FGF-basic), and vascular endothelial growth factor (VEGF) (Table 1). This association indicate a more inflammatory environment in samples with high proportion of innate immune cells. On the other hand, the proportion of T and B lymphocytes was significantly associated with fewer mediators, such as IP-10, IL-9, and RANTES, which correlates well with the recruitment and activation of adaptive immune cells (Table 1).

**TABLE 1:** Spearman’s correlation of inflammatory cells and mediators in MEE samples^*a*^

Mediator	Leukocyte population^*b*^								
	Neu	Mo-Macr	T cells	CD8^+^ Tc	CD4^+^ Th	CD4/CD8	B cells	NK	NKT
IL-8	0.551**	0.415*	0.061	0.144	−0.071	0.668***	0.062	−0.246	−0.372^*g*^
IL-1β	0.475*	0.553**	−0.008	0.040	−0.175	0.531*	0.021	−0.316	−0.224
IL-6	0.578***	0.440*	−0.015	0.118	0.041	0.568**	0.028	−0.251	−0.267
IL-10	0.429*	0.470*	0.292**	0.260	0.029	0.598**	0.286	−0.011	−0.038
TNF-α	0.224	0.512**	0.095	0.100	−0.031	0.265	0.112	−0.117	−0.069
IL-10/TNF	0.247	0.185	0.611	0.442	0.292	0.622**	0.628**	0.484*	0.176
IL-1ra	0.638***	0.496*	0.349	0.363	0.254	0.667**	0.348	0.028	−0.292
IL-2	0.529*	0.544**	0.245	0.241	0.107	0.568*	0.200	−0.120	−0.279
IL-4	0.503*	0.561**	0.301	0.233	0.109	0.566*	0.239	−0.047	−0.209
IL-5	0.458*	0.569**	0.196	0.115	0.054	0.371	0.281	−0.130	−0.315
IL-7	0.291	0.463*	0.431*	0.340	0.436	0.223	0.418^*e*^	0.394	−0.126
IL-9	0.250	0.318	0.798***	0.717***	0.715***	0.270	0.462*	0.679***	0.442*
IL-12	0.446*	0.614**	0.232	0.161	0.056	0.457^*d*^	0.279	−0.087	−0.311
IL-13	0.467*	0.407	−0.022	−0.082	−0.182	0.457^*d*^	0.052	−0.040	−0.290
IL-15	0.337	0.505*	0.180	0.060	−0.035	0.284	0.205	−0.167	−0.290
IL-17	0.522*	0.503*	0.265	0.245	0.088	0.564*	0.281	−0.031	−0.241
Eotaxin	0.450*	0.437*	0.344	0.346	0.196	0.603**	0.144	0.157	0.020
FGF-basic	0.517*	0.627**	0.345	0.181	0.095	0.547*	0.331	0.018	−0.258
G-CSF	0.583**	0.370	0.126	0.162	0.034	0.566*	0.152	−0.120	−0.306
GM-CSF	0.353	0.468*	0.218	0.093	0.032	0.415	0.176	−0.116	−0.394
IFN-γ	0.472*	0.433*	0.346	0.160	0.258	0.365	0.410^*f*^	−0.010	−0.420^*g*^
IP-10	0.277	0.322	0.380	0.520*	0.731***	−0.005	0.074	0.206	0.151
MCP-1	0.147	0.255	0.310	0.233	0.392	0.061	0.105	0.023	−0.026
MIP-1α	0.504*	0.459*	0.146	0.193	0.032	0.576*	0.154	−0.227	−0.349
PDGF-bb	0.276	0.475*	0.519*	0.415	0.465^*c*^	0.096	0.228	0.513*	0.359
MIP-1β	0.563**	0.585**	0.262	0.140	0.043	0.462^*e*^	0.316	−0.034	−0.270
RANTES	0.126	0.268	0.599**	0.526*	0.510*	0.098	0.240	0.614**	0.436*
VEGF	0.563**	0.543*	0.176	0.193	0.097	0.570*	0.252	−0.186	−0.367
M-CSF	0.442*	0.175	0.226	0.442	0.306	0.289	−0.045	0.038	0.143
IL-18	0.585**	0.312	0.295	0.295	0.296	0.475*	0.256	0.072	−0.118

a The potential correlation between inflammatory cells and mediators in MEE samples was assessed by Spearman’s *r* correlation.

b Significant *R* values are indicated in bold. *, *P *< 0.05; **, *P *< 0.01; ***, *P *< 0.001. Neu, neutrophils (CD66b^+^); Mo-Macr, monocytes/macrophages (CD14^+^ HLA-DR^+^); T cells, CD3^+^; CD8^+^, cytotoxic T cells; CD4^+^, T helper cells; CD4/CD8, % CD4^+^ cells/% CD8^+^ cells; B cells, CD19^+^; NK cells, CD56^+^ CD3^−^; NKT cells, CD56^+^ CD3^+^.

c *P* = 0.052.

d *P* = 0.057.

e *P* = 0.053.

f *P* = 0.058.

g *P* = 0.051.

When analyzing the potential correlations in NPH samples, however, no significant correlations between immune cell populations and inflammatory mediators were observed, except for a positive correlation between the proportion of natural killer T (NKT) cells (CD56^+^ CD3^+^ cells) and the concentrations of the proinflammatory mediator IL-1β (see Table S5 in the supplemental material), suggesting that the NPH environment is more homogenous between individuals.

### The relationship between the inflammatory responses or the microbiota profiles and clinical parameters.

In an attempt to address how differences in inflammatory responses between boys and girls relate to clinical parameters, we further investigated the following: we observed no difference in age, symptoms, or other clinical findings between boys and girls, including that they all suffered from conductive hearing loss due to confirmed OME at the time of inclusion (Table S1). However, among the five patients that had previous myringotomy with ventilation tube insertion, four (80%) were boys, indicating that the gender-based differences in inflammatory markers may also relate to the chronicity of OME (Table S1). Furthermore, to evaluate the potential differences in the microbiota composition in relation to specific clinical parameters we performed permutational multivariate analysis of variance (PERMANOVA). A significant difference in the microbiota structure was found between niches (see Table S6 in the supplemental material). However, there were no differences with regard to age at myringotomy, gender, number of affected ears (uni- or bilateral OME), or inflammation type (predominance of neutrophils or predominance of lymphocytes in the samples; Table S6). The last may be related to the low sample number, as inflammatory cell composition was only analyzed in 26 samples.

### Niche-driven associations between biomarker species and inflammatory mediators.

**FIG 4:**
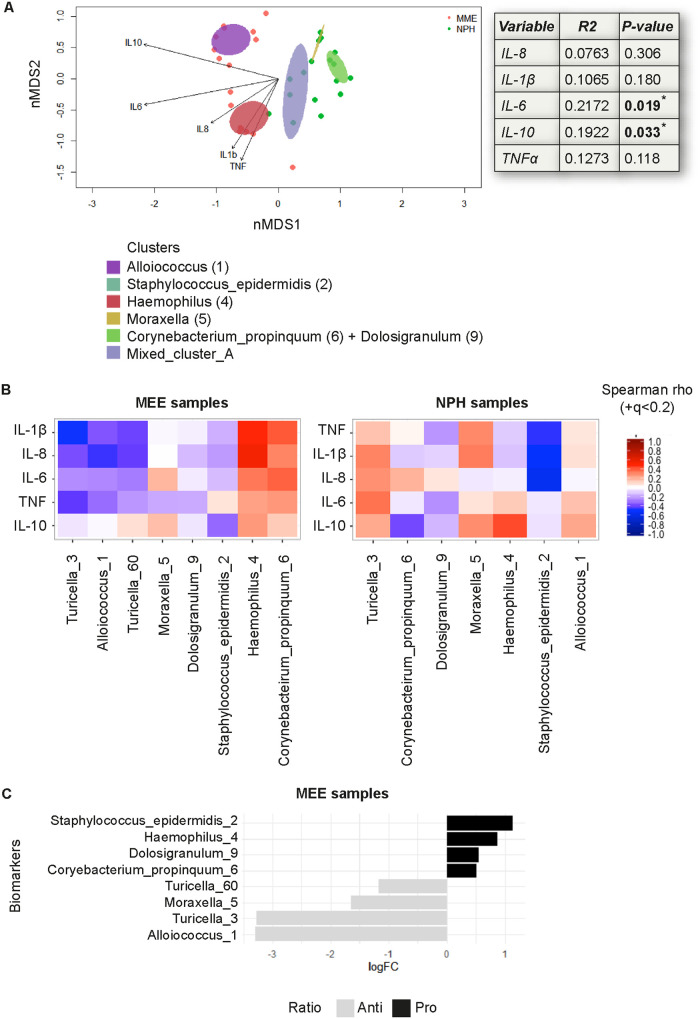
A niche-associated switch of the inflammatory profile in relation to specific biomarker species. The concentration of indicated cytokines assessed by CBA were correlated with the identified biomarker species. (A) An nMDS plot showing individual samples and clusters with arrows, with a length proportional to the correlation between the variable and the nMDS ordination (“envfit” from the *vegan* package). Linear correlation coefficient and *P* value, assessed by permutation (999) of environmental variables and corrected by niche, indicated for each mediator. (B) Heatmap shows Spearman’s correlation coefficients between indicated mediator and biomarker species in MEE (left) and NPH (right) samples. A switch in inflammatory profile in relation to specific biomarker species is seen between the niches. (C) Associations between anti- and proinflammatory responses (IL-10/TNF ratio) in MEE samples and biomarker species were assessed with the *metagenomeSeq* package. Strong associations with a log fold change of >2, were found, although these were not significant.

Finally, we investigated the relationship between the overall microbial community structure and the inflammatory mediators (assessed by CBA) in MEE and NPH samples by fitting the inflammatory profiles onto the microbiota nonmetric multidimensional scaling (nMDS) ordination. We found that IL-6 and IL-10 were significantly correlated with the nMDS ordination of the microbial community structure after correcting for niche (*envfit*; *R*^2^ = 0.22, *P* = 0.019 and *R*^2^ = 0.19, *P* = 0.033, respectively). Increasing IL-6 and IL-10 values were represented by two vectors pointing to the left of the nMDS ordination, with IL-6 pointing toward the *Haemophilus* (4) cluster and IL-10 pointing toward the *Alloiococcus* (1) cluster (Fig. 4A). The remaining inflammatory mediators were not significantly correlated with the nMDS ordination of the microbial community structure (Fig. 4A).

Interestingly, when separately analyzing the correlation between specific biomarker species and cytokines in MEE samples and NPH samples, there were strong (albeit not significant) niche-dependent associations (Fig. 4B). In MEE samples, *Haemophilus* (4) was strongly associated with the proinflammatory mediators IL-1β, IL-8, and IL-6, whereas in NPH samples *Haemophilus* (4) was more strongly associated with IL-10 and was inversely correlated with TNF-α, IL-1β, and IL-8, indicating a proinflammatory association in MEE samples and an anti-inflammatory profile in NPH samples (Fig. 4B). Similarly, in MEE samples, *Turicella* (3) and *Alloiococcus* (1) were inversely correlated with proinflammatory mediator IL-1β and TNF-α, whereas in NPH samples, these species were positively correlated with proinflammatory mediators (Fig. 4B).

Next, we further analyzed the inflammatory environment in the MEE samples, specifically, in relation to the biomarker species. When analyzing the IL-10/TNF ratio as a proxy for the predominance of anti- versus proinflammatory responses, we observed a trend, albeit not significant, toward an association between the anti- to proinflammatory ratio and biomarkers after correcting for multiple testing (Fig. 4C). A borderline significant association was found between *Alloiococcus* (1) and anti-inflammatory responses in MEE samples, which was lost after correcting for multiple testing (*P* = 0.056 and adjusted *P* value [*P*-adj] = 0.44; Fig. 4C), supporting the correlation with anti-inflammatory mediators in this niche in the analysis above. *Turicella* (3), *Moraxella* (5), and *Turicella* (60) were similarly associated with a higher IL-10/TNF ratio, whereas S. epidermidis (2), *Haemophilus* (4), *Dolosigranulum* (9), and *C. propinquum* (6) were associated with a lower ratio, albeit not significantly (Fig. 4C).

**FIG 5:**
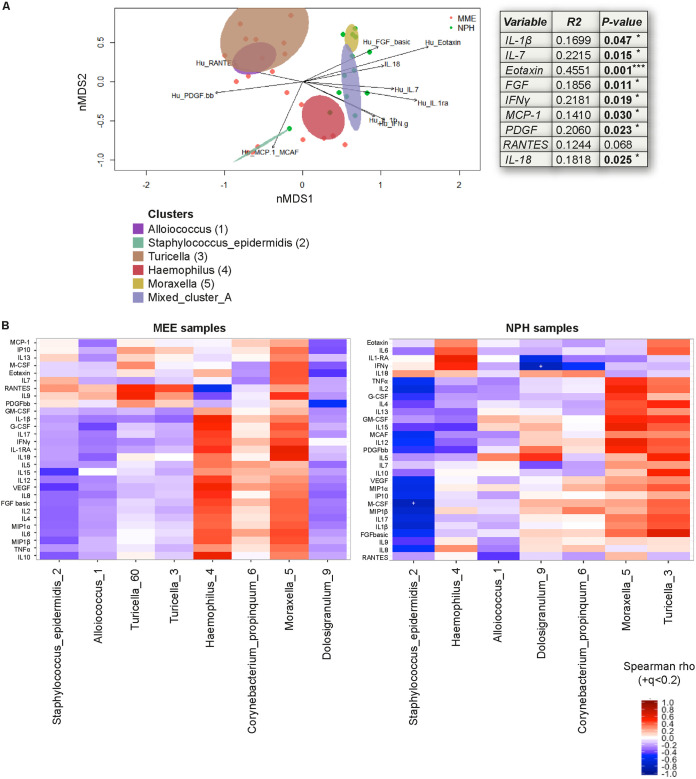
Inverse correlation between S. epidermidis and M-CSF and between *Dolosigranulum* and IFN-γ in NPH samples. The concentrations of indicated cytokines, assessed by Bio-Plex, were correlated with the identified biomarker species (see Fig. 3). (A) An nMDS plot showing individual samples and clusters, with arrows with a length proportional to the correlation between the variable and the nMDS ordination (“envfit” from the *vegan* package). Linear correlation coefficient and *P* value, assessed by permutation (999) of environmental variables and corrected by niche, indicated for each mediator. (B) Heatmap shows Spearman’s correlation coefficients between indicated mediator and biomarker species in MEE (left) and NPH (right) samples. A *q* value of <0.2 is indicated with a plus sign (+). A switch in inflammatory profile in relation to specific biomarker species is seen between the niches, with significant inverse correlations between S. epidermidis (2) and M-CSF and between *Dolosigranulum* (9) and IFN-γ in NPH samples specifically.

Expanding on these results, we analyzed the relationship between a panel of inflammatory mediators (assessed by Bio-Plex) and the overall microbiota composition. Only mediators that significantly explained the nMDS ordination of the microbiota are shown. RANTES, PDGFbb, and MCP-1 vectors pointed toward the left side of the nMDS where the samples that belong to *Alloiococcus* (1), *Turicella* (3), and S. epidermidis (2) clusters localized, whereas IL-1β, IL-7, eotaxin, FGF-basic, gamma interferon (IFN-γ), and IL-18 pointed toward the right side of the nMDS, where samples from *Haemophilus* (4), *Moraxella* (5), and Mixed_cluster_A clusters were located (Fig. 5A). Therefore, our data showed that the inflammatory response was strongly associated with the structure of the NPH and MEE microbial communities. When analyzing the correlations in MEE and NPH samples separately, a nonsignificant positive correlation with most inflammatory mediators investigated was seen in MEE samples for *Moraxella* (5) and *Haemophilus* (4), whereas a negative correlation was seen for S. epidermidis (2), *Alloiococcus* (1), and *Dolosigranulum* (9) (Fig. 5B, left). In NPH samples, S. epidermidis (2) was significantly and inversely correlated with macrophage colony-stimulating factor (M-CSF) and *Dolosigranulum* (9) significantly and inversely associated with IFN-γ (Fig. 5B, right). Overall, and in contrast to the MEE samples, *Turicella* (3) and *Dolosigranulum* (9) were positively associated with most inflammatory mediators in the NPH samples, again indicative of a “switch” in immune responses at specific niches for specific species (Fig. 5B).

## DISCUSSION

In this study, we set out to comprehensively characterize the inflammatory response (immune cells and mediators) in relation to the microbiota in MEE and NPH samples from patients with OME and to compare those with clinical parameters. To our knowledge, this is also the first study analyzing the microbiota in MEE and NPH samples from Swedish children, a group that compared with other patient populations investigated is equally prone to develop OME as other children but where investigations can be performed without the confounding effects of antibiotic intervention. Children with OME in Sweden are never subjected to prophylaxis or treatment with antibiotics. Also, children with sporadic AOM between ages 1 and 12 years are primarily recommended treatment with watchful waiting, which reduces the intake of antibiotics in possible study subjects (25). For comparison, Australian Indigenous children are prone to both chronic suppurative otitis media (CSOM) and OME and are routinely recommended treatment with amoxicillin instead of watchful waiting for AOM (15).

When analyzing the microbiota in MEE, EEC lavage, and NPH samples, the diversity of microbial species was significantly higher in the NPH compared to that in the other sites. Differences in microbiota profiles in these three environments have been detected in previous studies that suggested that both the EEC and the NPH served as potential reservoirs for the microbiota in the ME (26). Interestingly, in this study the MEE and EEC lavage samples were remarkably similar, with the microbiota profile in MEE samples exhibiting only some overlap with NPH samples. Several plausible reasons for this could be mentioned. First, the Eustachian tube is frequently blocked in patients with OME, which may prevent microorganisms from ascending from the NPH. Second, despite our best efforts to sterilize the EEC after the lavage, we cannot fully rule out the possibility of some contamination from the EEC into the MEE samples. However, if this were the case, we would have expected minor contamination rather than close-to-identical profiles. Third, traditionally, a healthy tympanic membrane is considered impermeable, although some peptides have been suggested to transit across it (27). Such peptides are, needless to say, much smaller than microbes, and an exchange of specific species between the ME and EEC through an intact tympanic membrane therefore poses an interesting hypothesis, although it seems unlikely. However, one could also speculate that chronic inflammation in the ME could be perpetuated through the tympanic membrane and affect the epithelial status quo in the EEC, thus selecting for similar microbes in both environments. Fourth, at the time of myringotomy, the tympanic membrane was intact, and the majority of patients had no indications of previous perforations, which would rule against a major contamination. Eight patients (29.6%) in this study had previously known perforations/tympanocentesis, in their past medical history, although earlier self-healing of spontaneous perforations could have occurred that the parents did not recall. In conclusion, although various hypotheses for the similar microbiota profiles found in the MEE and EEC samples exist, the most probable explanation would be a tympanic membrane breach from an earlier infection that allowed for exchange between the two niches.

Among the identified species, *Alloiococcus*, S. epidermidis, and *Turicella* predominated in the MEE and EEC lavage samples compared to the NPH samples. This is in line with results of previous studies (8, 28). MEEs also displayed a higher relative abundance of *Haemophilus* compared to that in EEC lavages. H. influenzae is a commonly reported OM pathogen that is especially prevalent in chronic OME and recurrent AOM (29). S. epidermidis, however, is generally considered part of the normal flora and has been identified in cerumen and EEC of healthy individuals (30). This is in line with the inverse association of this species with inflammatory mediators in our study. However, S. epidermidis has also been reported in MEE from patients with OM (31), and lysates of S. epidermidis have been proposed to activate proinflammatory responses (NF-κB activity and IL-8 release) and mucin expression *in vitro* (31). *Alloiococcus otitidis* is a potential emerging pathobiont in OM that has previously been reported in MEEs from patients prone to chronic suppurative otitis media (CSOM) (10, 11, 32), as well as in OME patients without a history of ear infections (20, 33), but also in the EEC of healthy individuals (30). Similarly, Turicella otitidis has previously been identified in MEE from patients with AOM (8), as well as in the EEC of healthy subjects (30). The role of these bacterial species as OM pathogens is still under debate (15, 34). Since many of the reported species are anaerobes, the build-up effusions in the ME during OME could potentially create local environments in which these organisms get an opportunity to thrive. Studies in rats and *in vitro* have indicated that A. otitidis elicits mild responses in the ME (33), yet is capable of inducing IL-12 release from monocytes (35) and activating lymphocytes (36). Based on their poor detection by culture, in more recent clinical studies, *A. otitidis* and *T. otitidis* have both been identified by PCR as the only organism in MEE samples from children with AOM and children with OME, suggesting that these species can act as pathogens alone (12, 37, 38). We found an inverse correlation between *Alloiococcus* and, among others, IL-12 in the MEE samples of our patients, which correlate well with the previously measured low inflammatory response in the rat model of OM (33). Similarly, *Turicella* was inversely correlated with proinflammatory mediators in the MEE samples. However, a strong, albeit not significant, positive association was seen with T lymphocyte-associated factors such as RANTES and IL-9, possibly indicating more adaptive T-cell responses in these samples. Thus, despite their described role as sole pathogens in OM in children, *Alloiococcus* and *Turicella* seem to evade proinflammatory responses, which warrants further in-depth analyses to better understand their role as emerging OM pathogens, as well as the underlying mechanisms.

What perpetuates the inflammation in OME, and what role specific bacteria play during the persisting inflammation, has long been unclear. The MEEs analyzed in this study were often devoid of or had very low concentrations of bacterial DNA. Thus, one possibility is that mucosal epithelial cells, in collaboration with already recruited leukocytes, perpetuate the inflammation even after the bacteria have been fully eliminated, suggesting an altered regulation of the immune system. Regulatory T cells have, for instance, been proposed to accumulate and prolong bacterial persistence in the ME in a mouse model of chronic OM (39). Another possible explanation is an underlying, undetected viral infection. Respiratory viruses play an important role in the onset of AOM, whereas their role in OME is debated (40). Due to the study setup and sample limitations, we were not able to analyze the presence of respiratory viruses in this study. At the time of myringotomy, which is performed during general anesthesia, children with symptoms of an ongoing acute upper respiratory tract infection, viral or bacterial, were not accepted for surgery. Thus, all samples were obtained from children without any apparent simultaneous, symptomatic infection. Nevertheless, this does not rule out the possibility of a subclinical infection and warrants further investigations of the role of respiratory viruses on microbiota profiles and inflammation during OME. We did find inflammatory cells and mediators involved in antiviral immune responses, such as interferons, NK cells, and CD8^+^ cytotoxic T lymphocytes. Even so, these are also involved in immune responses against, e.g., nontypeable Haemophilus influenzae (41). In this population, MEE samples were geared toward a more innate immune profile, with neutrophils being the predominant immune cell population present in all MEE samples and IL-6 being present at higher levels than in the NPH samples. Conversely, NPH samples had a higher proportion of T and B lymphocytes and showed higher levels of IL-1β and TNF-α. Interestingly, we found both a niche- and gender-dependent association with inflammatory cells and mediators. Although still displaying a primarily neutrophil-rich environment, MEE samples, specifically those from female patients, exhibited a higher prevalence of T lymphocytes (CD4^+^ as well as CD8^+^) and significantly higher concentrations of most inflammatory mediators investigated. This finding is difficult to conclusively evaluate in this specific cohort since the endpoint for all patients was treatment with ventilating tubes without correlation to gender. However, these gender differences in the immune response in OME may be of interest since myringotomy with ventilation tube insertion is more common among boys (57 to 60%) than among girls (40 to 43%) in the United States, as well as in Sweden (42, 43). Furthermore, complications to AOM are somewhat more common in boys in many studies (44–46). Among these studies, only two investigated inflammatory markers (CRP, fever, or white blood cell counts), and neither did so in relation to gender (45, 46). However, other studies have reported that CD8^+^ cytotoxic T lymphocytes, CD4^+^ T helper cells, and mediators such as IL-1, TNF-α, IL-2, and IL-12 are involved in protection against potential pathogens (41, 47, 48). Thus, it is tempting to speculate that the gender-based differences in inflammatory markers may relate to the chronicity of OME. It is also interesting to note that otitis-prone children have been shown to display significant downregulation of several factors involved in innate immune responses, further emphasizing the importance of well-regulated immune responses (22).

Innate immune cells (such as neutrophils and monocytes-macrophages) are among the first leukocytes to be recruited to sites of infection, but they were observed in this study to clearly predominate and associate with a higher degree of inflammation in the chronic situation of OME as well. The innate immune cell predominance significantly correlated with a vast array of inflammatory mediators. Pro- and anti-inflammatory cytokines, as well as proangiogenic factors and factors associated with tissue remodeling, were significantly correlated with higher levels of neutrophils and monocytes-macrophages, indicating a more active inflammatory environment in these MEEs. Inflammatory mediators have previously been shown to accumulate over time in the MEE during OME (49). Several factors identified in this study, such as granulocyte colony-stimulating factor (G-CSF), granulocyte-macrophage colony-stimulating factor (GM-CSF), and M-CSF, are well-known survival factors for myeloid cells (50), which may explain the predominance of innate myeloid cells in the MEE samples. T lymphocytes, on the other hand, significantly correlated with a specific set of mediators, including IL-9, IP-10, and RANTES. Whereas RANTES and IP-10 are well-known chemoattractants for, e.g., activated T lymphocytes, IP-10 has been related to IFN-γ and Th1 signaling and less is known regarding IL-9 (22, 51, 52). A recently identified subpopulation of T lymphocytes (Th9 cells) has been identified as a major source for this pleiotropic cytokine, possibly explaining this association (53).

Interestingly, our results also indicated a niche-dependent inflammatory profile in relation to specific biomarker species. *Haemophilus* was strongly associated with proinflammatory mediators in MEE, in line with its role as an otopathogen. In the NPH samples, however, we observed that the presence of *Haemophilus* was rather associated with the anti-inflammatory mediator IL-10 and was inversely correlated with proinflammatory mediators, indicative of pro- and anti-inflammatory profiles in the ME and NPH, respectively. Conversely, *Turicella* and *Alloiococcus* were inversely and positively correlated with proinflammatory mediators in MEE and the NPH, respectively. This is in line with previous observations that *Alloiococcus* elicits mild responses in the ME (33), yet is somewhat contradictory to their roles as potential pathogens (12, 37, 38). However, it is possible that the association with an anti-inflammatory profile in MEE facilitated persistence of the bacteria and consequently prolonged low-grade inflammation in the ME. This will be interesting to investigate further in the future. Furthermore, the relative abundances of these species are different between the niches, which may affect the inflammatory responses at these sites. Alternatively, niche-specific inflammatory responses induced by various organisms may drive their persistence or elimination in that niche. Previous studies have reported that the expression and distribution of pattern recognition receptors (PRRs) differs between anatomical sites. For instance, Toll-like receptor 4 (TLR4) expression was found to be higher at the distal end than at the proximal end of the Eustachian tube, where colonization is low and high, respectively (54–56). Thus, differential expression of PRRs at the specific niches may well contribute significantly to and explain the pro- and anti-inflammatory profiles in the ME and NPH for pathogens. Recently, Man et al. described how the loss of topography between the nasopharyngeal and oral niche seems to precede the development of respiratory tract infections and is driven by a proportional influx of oral taxa in the nasopharynx (57). The role of inflammation in this process is not known. Potentially, during an inflammatory episode, there is a loss of upper respiratory microbial topography, and this might be linked to our observation of niche-dependent distribution of certain taxa with different inflammatory profiles. For instance, *C. propinquum*, a bacterium more prevalent in the NPH than in the ME and commonly associated with a lower incidence of respiratory tract infections (58), was associated with more anti-inflammatory profiles in the NPH and proinflammatory profiles in the MEE. The opposite is true for organisms such as *Turicella* and *Alloiococcus* that are almost exclusively found in the ME and EEC (8, 28), which are correlated inversely with proinflammatory mediators in MEE, whereas a positive association was seen in NPH samples. The presence of these and other bacterial species in the nonpreferred niche, in combination with site-specific environmental factors and signals from the local microbiota, may induce and thus correlate with opposite immune responses (pro- or anti-inflammatory) compared to their preferred niche. To our knowledge, this is the first study reporting this niche-dependent variation in inflammatory profile in relation to specific species, which will be of interest to look into further in the future.

To summarize, this study presents evidence of niche-specific immune reactions in relation to both gender and specific microorganisms in patients with OME. Although the clinical implications are limited by the small sample size, the results are nevertheless hypothesis generating and indicate site-specific immune responses associated with prevalence of specific microorganisms in the ME and NPH, where, e.g., the presence of *Haemophilus* spp. in MEE is associated with IL-1β, IL-8, and IL-6, whereas an association with IL-10 was observed in NPH samples. Conversely, *Turicella* and *Alloiococcus* were inversely correlated with IL-1β and TNF-α when present in MEE samples, whereas their presence in NPH samples was positively associated with these mediators. Furthermore, the higher prevalence of T lymphocytes and inflammatory mediators observed in MEE from female patients may mediate a more rapid disease resolution and shed light on the gender-associated prevalence and severity of OM. A better understanding of the interplay between specific bacteria and the host’s immune response and other host parameters can be used to delineate the underlying mechanisms of OME pathogenesis and has the potential to provide novel diagnostic and preventive strategies in the future.

## MATERIALS AND METHODS

### Patient inclusion.

According to Swedish guidelines on OME, patients with persistent bilateral hearing loss for a minimum of 3 months and clinical findings of OME will be offered treatment with bilateral myringotomy and insertion of ventilation tubes. All such patients, scheduled for surgery at Ängelholm Hospital, Sweden, between December 2016 until April 2018, were asked to participate in this prospective study (see Fig. S1A in the supplemental material for the inclusion scheme). Patients with recent acute otitis media, immunological disorders, or other severe diseases demanding a higher level of postoperative surveillance than that possible at the operation ward were excluded. OME is never treated with antibiotics in Sweden, and none of the included patients received any antibiotics just prior to the myringotomy. A total of 36 children (18 boys [50%] and 18 girls [50%]) with a median age of 61 months (range, 24 to 135 months) were enrolled. Among these, nine patients (three boys [33%] and six girls [67%]) were excluded due to bilateral dry ears. The median age of the excluded patients was 70 months. See Fig. S1 for overview of the inclusion scheme and Table S1 for characteristics of the included patients. Study permission was obtained from the Research Ethics Committee in Lund (Dnr 2012/11), and all work was conducted in accordance with the Declaration of Helsinki. Written informed consent was obtained from each child’s legal guardians.

### Sample collection and preparation.

Prior to myringotomy, the ears were rinsed from cerumen and flushed with 1 ml sterile saline. This external ear canal (EEC) lavage fluid was collected for microbiome analyses. The ear canals were subsequently washed once with 70% denatured alcohol. After incision of the tympanic membrane, the middle ear effusion (MEE) was aspirated into a sterile trap, and 1 ml sterile saline was used to clear the suction catheter. Nasopharyngeal (NPH) swabs were collected and swirled in 1 ml sterile saline. See Fig. S1B for a summary of sample collection procedure. Besides use for the analyses in this study, all MEE and NPH samples were also routinely sent to the Department of Clinical Microbiology, Skåne University Hospital, for identification of bacteria.

Aliquots of all samples were immediately frozen at −80°C for subsequent microbiome analyses. Cells were collected by centrifugation at 350 × *g* for 10 min. The supernatants were collected and stored at −80°C until used for analyses of inflammatory mediators. Red blood cells in the cell pellets were lysed using ammonium-chloride-potassium (ACK) lysing buffer (Thermo Fisher Scientific). The cells were washed, and the total number of cells in the samples, as well as the percentage of viable cells, was calculated by the trypan blue exclusion assay.

### Flow cytometry.

Freshly isolated cells were stained with relevant antibodies for a total of 20 min at room temperature and analyzed by flow cytometry. Due to various sample amounts (number of cells in each sample), we were not able to perform all analyses on all samples and patients. The antibodies (with dilutions in parenthesis) used were as follows: CD14-fluorescein isothiocyanate (FITC) clone M5E2 (1:10), HLA-DR-phycoerythrin (PE) clone G46-6 (1:20), HLA-DR-allophycocyanin (APC) clone L243 (1:50), CD86-PE clone IT2.2 (1:20), CD33-APC clone WM53 (1:10), CD3-FITC clone HIT3a (1:25), CD4-APC clone RPA-T4 (1:25), CD8-PE HIT8a (1:25), CD8-APC clone RPA-T8 (1:25), CD25-FITC clone 2A3 (1:10), CD127-PE clone HIL-7R-M21 (1:20), CD45RA-FITC clone HI100 (1:10), CD45RO-PE clone UCHL1 (1:10), CD19-PE clone HIB19 (1:20), CD138-APC clone MI15 (1:15), CD183-PE clone 1C6/CXCR3 (1:25), EpCAM-FITC clone EBA-1 (1:25), CD66b-PE clone G10F5 (1:20), CD64-APC clone 10.1 (1:20), CD294-FITC clone BM16 (1:25), CD209-APC clone DCN46 (1:15), CD203c-PE clone NP4D6 (1:25), CD196-PE clone 11A9 (1:20), CD161-FITC clone DX12 (1:10), and CD56-APC clone B159 (1:10), all from BD Biosciences. Cells were analyzed using an FACSCalibur flow cytometer (BD Biosciences, San Jose, CA). All analyses were performed using 7-AAD dead exclusion stain (BD Biosciences).

### Analyses of inflammatory mediators.

After thawing on ice, MEE and NPH cell-free samples were analyzed using Bio-Plex and a cytometric cytokine bead array (CBA). (i) For Bio-Plex analysis, all samples were supplemented with 0.5% bovine serum albumin (BSA) (from a 10% stock) before analysis. The Bio-Plex Pro Human Cytokine 27-plex assay (Bio-Rad Laboratories) with two additional analytes was used according to the manufacturer’s instructions. The entire panel includes FGF-basic, eotaxin, G-CSF, GM-CSF, IFN-γ, IL-1β, IL-1ra, IL-2, IL-4, IL-5, IL-6, IL-7, IL-8, IL-9, IL-10, IL-12 (p70), IL-13, IL-15, IL-17A, IP-10, MCP-1 (MCAF), MIP-1α, MIP-1β, PDGF-BB, RANTES, TNF-α, and VEGF, with M-CSF and IL-18 added separately to the panel. (ii) The levels of IL-8, IL-1β, IL-6, IL-10, TNF-α, and IL-12p70 were also assessed using a human inflammatory cytokine cytometric bead array (detection limits = 3.6, 7.2, 2.5, 3.3, 3.7, and 1.9 pg/ml, respectively; BD Biosciences, San Diego, CA). IL-12p70 was largely undetectable by this method and hence was excluded from further analyses.

### 16S rRNA sequencing and bioinformatic processing.

Bacterial DNA of paired MEE, NPH, and EEC lavages was isolated and quantified as previously described (59, 60). In total, 24 MEE samples out of 54, 20 NPH out of 27, and 30 EEC out of 38 fulfilled our quality control standards for reliable analyses. Only samples with a bacterial density of at least 0.1 pg/μl above the background (DNA quantity of the negative controls) as measured with real-time PCR were considered for sequencing. Amplification of the V4 hypervariable region of the 16S rRNA gene, library preparation, and sequencing were executed as previously described (61). Sequenced amplicons were processed using a bioinformatics pipeline that included trimming, error correction, assembly, and 97% identity clustering of reads into operational taxonomic units (OTUs). After removal of chimeric reads, OTUs were taxonomically annotated using the SILVA database (61). To avoid OTUs with identical annotations, we referred to OTUs using their taxonomical annotations combined with a rank number based on the abundance of each given OTU (61). The frequency-based method from *decontam* R package (62) was used to identify and remove procedural contaminating OTUs in the samples. After removing contaminating OTUs, the rarefied “OTU-counts” table was used for calculations of α-diversity. The “OTU-proportions” table was used for all other downstream analyses, including β-diversity and hierarchical clustering.

### Statistical analyses.

Statistical analyses on immunological parameters were performed using Prism v8 (GraphPad, Inc., San Diego, CA). Data from two groups were compared by the Mann-Whitney U test, whereas data comparing more than two groups were analyzed by Kruskal-Wallis with Dunn’s multiple-comparison test. The nonparametric Spearman’s test was used for rank correlation analysis. A *P* value of <0.05 was considered significant.

For the analysis of the microbiome and its correlation with the immune response, we used R version 3.5 (R Core Team) within RStudio v1.2, primarily with the packages *vegan* (63), *phyloseq* (64), *metagenomeSeq* (65), *microbiome* (66), and *ggplot2* (67). Benjamini-Hochberg (BH) adjusted *P* values (*q* values) were generated where appropriate. A *P* value and a *q* value of 0.05 was considered significant, unless otherwise stated.

α-Diversity was estimated by observed number of OTUs and by the Shannon diversity index; the latter takes into account both species richness and evenness of the samples. Statistical significance of the differences in α-diversity was calculated using the pairwise nonparametric Mann-Whitney U test corrected for multiple comparison. A nonmetric multidimensional scaling (nMDS) plot and hierarchical clustering dendrogram based on the Bray-Curtis dissimilarity matrix were used to visualize differences in the overall microbial community composition between niches. We performed PERMANOVA tests using the “adonis” function (*vegan* package, R), to test for the significance of niche and other environmental factors (i.e., gender, age, inflammation type, fluid description, affected ears, and etiology) on the overall microbiota composition. The optimal number of clusters was estimated using the average silhouette width and the Calinski-Harabasz index (*fpc* R package). To confirm with an unsupervised quantitative method which biomarker OTUs were associated with the clusters, we used a random forest algorithm (*VSURF* R package). Associations between the biomarker species abundance and either the NPH niche or the ME niche, as well as associations between biomarkers and anti- to proinflammatory ratio, were assessed using the *metagenomeSeq* package. Associations between the biomarkers and clusters were assessed on the log-transformed relative abundance of the biomarkers by linear models, corrected by niche, and followed by Tukey’s *post hoc* test. The “envfit” function from the *vegan* package was used to fit the immune response through the expression of inflammatory mediators onto the microbiota nMDS ordination. This function performs multivariate analysis of variance (MANOVA) and linear correlations for categorical and continuous variables, respectively. The significance of fitted vectors was assessed using permutation (999) of environmental variables and corrected by niche. We used a Spearman’s rank correlation to determine any correlations between cytokines and cluster biomarkers and the “correlation.heatmap” function from the microbiome R package for visualization. For Spearman’s rank correlations, a *q* value of ≤0.2 was considered significant (68).

## Supplementary Material

Supplemental file 1

## References

[1] Monasta L, Ronfani L, Marchetti F, Montico M, Vecchi Brumatti L, Bavcar A, Grasso D, Barbiero C, Tamburlini G 2012 Burden of disease caused by otitis media: systematic review and global estimates. PLoS One 7:e36226. doi:10.1371/journal.pone.0036226. 22558393 PMC3340347

[2] Qureishi A, Lee Y, Belfield K, Birchall JP, Daniel M 2014 Update on otitis media—prevention and treatment. Infect Drug Resist 7:15–24. doi:10.2147/IDR.S39637. 24453496 PMC3894142

[3] Simon F, Haggard M, Rosenfeld RM, Jia H, Peer S, Calmels MN, Couloigner V, Teissier N 2018 International consensus (ICON) on management of otitis media with effusion in children. Eur Ann Otorhinolaryngol Head Neck Dis 135:S33–S39. doi:10.1016/j.anorl.2017.11.009. 29398506

[4] Kubba H, Pearson JP, Birchall JP 2000 The aetiology of otitis media with effusion: a review. Clin Otolaryngol Allied Sci 25:181–194. doi:10.1046/j.1365-2273.2000.00350.x. 10944048

[5] Mittal R, Lisi CV, Gerring R, Mittal J, Mathee K, Narasimhan G, Azad RK, Yao Q, Grati M, Yan D, Eshraghi AA, Angeli SI, Telischi FF, Liu XZ 2015 Current concepts in the pathogenesis and treatment of chronic suppurative otitis media. J Med Microbiol 64:1103–1116. doi:10.1099/jmm.0.000155. 26248613 PMC4835974

[6] Bergenfelz C, Hakansson AP 2017 *Streptococcus pneumoniae* otitis media pathogenesis and how it informs our understanding of vaccine strategies. Curr Otorhinolaryngol Rep 5:115–124. doi:10.1007/s40136-017-0152-6. 28616365 PMC5446555

[7] Francois M 1997 New views on the pathogenesis of acute otitis media and its complications. Clin Microbiol Infect 3(Suppl 3):S5–S12. 11869222

[8] Man WH, van Dongen TMA, Venekamp RP, Pluimakers VG, Chu M, van Houten MA, Sanders EAM, Schilder AGM, Bogaert D 2019 Respiratory microbiota predicts clinical disease course of acute otorrhea in children with tympanostomy tubes. Pediatr Infect Dis J 38:e116–e125. doi:10.1097/INF.0000000000002215. 30299424

[9] Stol K, Verhaegh SJ, Graamans K, Engel JA, Sturm PD, Melchers WJ, Meis JF, Warris A, Hays JP, Hermans PW 2013 Microbial profiling does not differentiate between childhood recurrent acute otitis media and chronic otitis media with effusion. Int J Pediatr Otorhinolaryngol 77:488–493. doi:10.1016/j.ijporl.2012.12.016. 23369612 PMC7132406

[10] Jervis-Bardy J, Rogers GB, Morris PS, Smith-Vaughan HC, Nosworthy E, Leong LE, Smith RJ, Weyrich LS, De Haan J, Carney AS, Leach AJ, O’Leary S, Marsh RL 2015 The microbiome of otitis media with effusion in Indigenous Australian children. Int J Pediatr Otorhinolaryngol 79:1548–1555. doi:10.1016/j.ijporl.2015.07.013. 26228497

[11] Chan CL, Wabnitz D, Bardy JJ, Bassiouni A, Wormald PJ, Vreugde S, Psaltis AJ 2016 The microbiome of otitis media with effusion. Laryngoscope 126:2844–2851. doi:10.1002/lary.26128. 27335217

[12] Holder RC, Kirse DJ, Evans AK, Whigham AS, Peters TR, Poehling KA, Swords WE, Reid SD 2015 Otopathogens detected in middle ear fluid obtained during tympanostomy tube insertion: contrasting purulent and non-purulent effusions. PLoS One 10:e0128606. doi:10.1371/journal.pone.0128606. 26039250 PMC4454603

[13] Farajzadah Sheikh A, Saki N, Roointan M, Ranjbar R, Yadyad MJ, Kaydani A, Aslani S, Babaei M, Goodarzi H 2015 Identification of *Alloiococcus otitidis*, *Streptococcus pneumoniae*, *Moraxella catarrhalis* and *Haemophilus influenzae* in children with otitis media with effusion. Jundishapur J Microbiol 8:e17985. doi:10.5812/jjm.17985. 25861433 PMC4386075

[14] Verhoeff M, van der Veen EL, Rovers MM, Sanders EA, Schilder AG 2006 Chronic suppurative otitis media: a review. Int J Pediatr Otorhinolaryngol 70:1–12. doi:10.1016/j.ijporl.2005.08.021. 16198004

[15] Jervis-Bardy J, Carney AS, Duguid R, Leach AJ 2017 Microbiology of otitis media in Indigenous Australian children: review. J Laryngol Otol 131:S2–S11. doi:10.1017/S0022215116009294. 28088924

[16] Bhutta MF, Thornton RB, Kirkham LS, Kerschner JE, Cheeseman MT 2017 Understanding the aetiology and resolution of chronic otitis media from animal and human studies. Dis Model Mech 10:1289–1300. doi:10.1242/dmm.029983. 29125825 PMC5719252

[17] Bernstein JM, Tsutsumi H, Ogra PL 1985 The middle ear mucosal immune system in otitis media with effusion. Am J Otolaryngol 6:162–168. doi:10.1016/s0196-0709(85)80079-x. 4040332

[18] Mittal R, Kodiyan J, Gerring R, Mathee K, Li JD, Grati M, Liu XZ 2014 Role of innate immunity in the pathogenesis of otitis media. Int J Infect Dis 29:259–267. doi:10.1016/j.ijid.2014.10.015. 25447732 PMC4310697

[19] Bernstein JM, Szymanski C, Albini B, Sun M, Ogra PL 1978 Lymphocyte subpopulations in otitis media with effusion. Pediatr Res 12:786–788. doi:10.1203/00006450-197807000-00009. 308634

[20] Val S, Poley M, Anna K, Nino G, Brown K, Perez-Losada M, Gordish-Dressman H, Preciado D 2018 Characterization of mucoid and serous middle ear effusions from patients with chronic otitis media: implication of different biological mechanisms? Pediatr Res 84:296–305. doi:10.1038/s41390-018-0060-6. 29915406 PMC6185811

[21] Verhoeven D, Nesselbush M, Pichichero ME 2013 Lower nasopharyngeal epithelial cell repair and diminished innate inflammation responses contribute to the onset of acute otitis media in otitis-prone children. Med Microbiol Immunol 202:295–302. doi:10.1007/s00430-013-0293-2. 23576001 PMC3943207

[22] Kaur R, Casey J, Pichichero M 2016 Differences in innate immune response gene regulation in the middle ear of children who are otitis prone and in those not otitis prone. Am J Rhinol Allergy 30:218–223. 28124644 10.2500/ajra.2016.30.4393 PMC5108842

[23] Sharma SK, Pichichero ME 2013 Cellular immune response in young children accounts for recurrent acute otitis media. Curr Allergy Asthma Rep 13:495–500. doi:10.1007/s11882-013-0370-z. 24022464 PMC3884676

[24] Johnson IJ, Brooks T, Hutton DA, Birchall JP, Pearson JP 1997 Compositional differences between bilateral middle ear effusions in otitis media with effusion: evidence for a different etiology? Laryngoscope 107:684–689. doi:10.1097/00005537-199705000-00024. 9149175

[25] Läkemedelsverket (Medical Products Agency Sweden). 2010 Diagnostics, treatment and monitoring of acute otitis media (AOM)—new recommendation. Information från Läkemedelsverket 5:13–24.

[26] Chan CL, Wabnitz D, Bassiouni A, Wormald PJ, Vreugde S, Psaltis AJ 2017 Identification of the bacterial reservoirs for the middle ear using phylogenic analysis. JAMA Otolaryngol Head Neck Surg 143:155–161. doi:10.1001/jamaoto.2016.3105. 27812691

[27] Kurabi A, Pak KK, Bernhardt M, Baird A, Ryan AF 2016 Discovery of a biological mechanism of active transport through the tympanic membrane to the middle ear. Sci Rep 6:22663. doi:10.1038/srep22663. 26946957 PMC4780071

[28] Kolbe AR, Castro-Nallar E, Preciado D, Pérez-Losada M 2019 Altered middle ear microbiome in children with chronic otitis media with effusion and respiratory illnesses. Front Cell Infect Microbiol 9:339. doi:10.3389/fcimb.2019.00339. 31637220 PMC6787523

[29] Ngo CC, Massa HM, Thornton RB, Cripps AW 2016 Predominant bacteria detected from the middle ear fluid of children experiencing otitis media: a systematic review. PLoS One 11:e0150949. doi:10.1371/journal.pone.0150949. 26953891 PMC4783106

[30] Stroman DW, Roland PS, Dohar J, Burt W 2001 Microbiology of normal external auditory canal. Laryngoscope 111:2054–2059. doi:10.1097/00005537-200111000-00035. 11801996

[31] Val S, Mubeen H, Tomney A, Chen S, Preciado D 2015 Impact of *Staphylococcus epidermidis* lysates on middle ear epithelial proinflammatory and mucogenic response. J Invest Med 63:258–266. doi:10.1097/JIM.0000000000000127. 25503091

[32] Ashhurst-Smith C, Hall ST, Walker P, Stuart J, Hansbro PM, Blackwell CC 2007 Isolation of *Alloiococcus otitidis* from Indigenous and non-Indigenous Australian children with chronic otitis media with effusion. FEMS Immunol Med Microbiol 51:163–170. doi:10.1111/j.1574-695X.2007.00297.x. 17666076

[33] Tano K, von Essen R, Eriksson PO, Sjöstedt A 2008 *Alloiococcus otitidis*—otitis media pathogen or normal bacterial flora? APMIS 116:785–790. doi:10.1111/j.1600-0463.2008.01003.x. 19024598

[34] Chan CL, Richter K, Wormald PJ, Psaltis AJ, Vreugde S 2017 *Alloiococcus otitidis* forms multispecies biofilm with *Haemophilus influenzae*: effects on antibiotic susceptibility and growth in adverse conditions. Front Cell Infect Microbiol 7:344. doi:10.3389/fcimb.2017.00344. 28824879 PMC5539592

[35] Himi T, Kita H, Mitsuzawa H, Harimaya A, Tarkkanen J, Hendolin P, Ylikoski J, Fujii N 2000 Effect of *Alloiococcus otitidis* and three pathogens of otitis media in production of interleukin-12 by human monocyte cell line. FEMS Immunol Med Microbiol 29:101–106. doi:10.1111/j.1574-695X.2000.tb01511.x. 11024348

[36] Tarkkanen J, Himi T, Harimaya A, Atshushi H, Carlson P, Ylikoski J, Mattila PS 2000 Stimulation of adenoidal lymphocytes by *Alloiococcus otitidis*. Ann Otol Rhinol Laryngol 109:958–964. doi:10.1177/000348940010901010. 11051437

[37] Holder RC, Kirse DJ, Evans AK, Peters TR, Poehling KA, Swords WE, Reid SD 2012 One third of middle ear effusions from children undergoing tympanostomy tube placement had multiple bacterial pathogens. BMC Pediatr 12:87. doi:10.1186/1471-2431-12-87. 22741759 PMC3475091

[38] Sillanpaa S, Kramna L, Oikarinen S, Sipila M, Rautiainen M, Aittoniemi J, Laranne J, Hyoty H, Cinek O 2017 Next-generation sequencing combined with specific PCR assays to determine the bacterial 16S rRNA gene profiles of middle ear fluid collected from children with acute otitis media. mSphere 2:e00006-17. doi:10.1128/mSphere.00006-17. 28357413 PMC5362748

[39] Hirano T, Kodama S, Kawano T, Suzuki M 2016 Accumulation of regulatory T cells and chronic inflammation in the middle ear in a mouse model of chronic otitis media with effusion induced by combined eustachian tube blockage and nontypeable *Haemophilus influenzae* infection. Infect Immun 84:356–364. doi:10.1128/IAI.01128-15. 26553466 PMC4694021

[40] Thornton RB, Hakansson A, Hood DW, Nokso-Koivisto J, Preciado D, Riesbeck K, Richmond PC, Su YC, Swords WE, Brockman KL 2020 Panel 7—Pathogenesis of otitis media—a review of the literature between 2015 and 2019. Int J Pediatr Otorhinolaryngol 130(Suppl 1):109838. doi:10.1016/j.ijporl.2019.109838. 31879085 PMC7062565

[41] King PT, Ngui J, Farmer MW, Hutchinson P, Holmes PW, Holdsworth SR 2008 Cytotoxic T lymphocyte and natural killer cell responses to non-typeable *Haemophilus influenzae*. Clin Exp Immunol 152:542–551. doi:10.1111/j.1365-2249.2008.03667.x. 18462210 PMC2453218

[42] Gisselsson-Solen M, Reference group for the National Quality Register for Tympanic Membrane Ventilation Tubes. 2018 The Swedish grommet register—hearing results and adherence to guidelines. Int J Pediatr Otorhinolaryngol 110:105–109. doi:10.1016/j.ijporl.2018.05.010. 29859568

[43] Keyhani S, Kleinman LC, Rothschild M, Bernstein JM, Anderson R, Chassin M 2008 Overuse of tympanostomy tubes in New York metropolitan area: evidence from five hospital cohort. BMJ 337:a1607. doi:10.1136/bmj.a1607. 18835846 PMC2563262

[44] Krishnan M, Walijee H, Jesurasa A, De S, Sinha A, Sharma R, Donne A 2020 Clinical outcomes of intracranial complications secondary to acute mastoiditis: the Alder Hey experience. Int J Pediatr Otorhinolaryngol 128:109675. doi:10.1016/j.ijporl.2019.109675. 31563751

[45] Mansour T, Yehudai N, Tobia A, Shihada R, Brodsky A, Khnifies R, Barzilai R, Srugo I, Luntz M 2019 Acute mastoiditis: 20 years of experience with a uniform management protocol. Int J Pediatr Otorhinolaryngol 125:187–191. doi:10.1016/j.ijporl.2019.07.014. 31369930

[46] Groth A, Enoksson F, Hultcrantz M, Stalfors J, Stenfeldt K, Hermansson A 2012 Acute mastoiditis in children aged 0–16 years—a national study of 678 cases in Sweden comparing different age groups. Int J Pediatr Otorhinolaryngol 76:1494–1500. doi:10.1016/j.ijporl.2012.07.002. 22832239

[47] Sharma SK, Roumanes D, Almudevar A, Mosmann TR, Pichichero ME 2013 CD4^+^ T-cell responses among adults and young children in response to *Streptococcus pneumoniae* and *Haemophilus influenzae* vaccine candidate protein antigens. Vaccine 31:3090–3097. doi:10.1016/j.vaccine.2013.03.060. 23632305 PMC3777711

[48] Juhn SK, Jung MK, Hoffman MD, Drew BR, Preciado DA, Sausen NJ, Jung TT, Kim BH, Park SY, Lin J, Ondrey FG, Mains DR, Huang T 2008 The role of inflammatory mediators in the pathogenesis of otitis media and sequelae. Clin Exp Otorhinolaryngol 1:117–138. doi:10.3342/ceo.2008.1.3.117. 19434244 PMC2671742

[49] Matkovic S, Vojvodic D, Baljosevic I 2007 Cytokine levels in groups of patients with different duration of chronic secretory otitis. Eur Arch Otorhinolaryngol 264:1283–1287. doi:10.1007/s00405-007-0373-2. 17643258

[50] Millrud CR, Bergenfelz C, Leandersson K 2017 On the origin of myeloid-derived suppressor cells. Oncotarget 8:3649–3665. doi:10.18632/oncotarget.12278. 27690299 PMC5356220

[51] Harimaya A, Fujii N, Himi T 2009 Preliminary study of proinflammatory cytokines and chemokines in the middle ear of acute otitis media due to *Alloiococcus otitidis*. Int J Pediatr Otorhinolaryngol 73:677–680. doi:10.1016/j.ijporl.2008.12.033. 19185927

[52] Liu M, Guo S, Hibbert JM, Jain V, Singh N, Wilson NO, Stiles JK 2011 CXCL10/IP-10 in infectious diseases pathogenesis and potential therapeutic implications. Cytokine Growth Factor Rev 22:121–130. doi:10.1016/j.cytogfr.2011.06.001. 21802343 PMC3203691

[53] Chen J, Guan L, Tang L, Liu S, Zhou Y, Chen C, He Z, Xu L 2019 T helper 9 cells: a new player in immune-related diseases. DNA Cell Biol 38:1040–1047. doi:10.1089/dna.2019.4729. 31414895 PMC6791470

[54] Munford RS 2008 Sensing gram-negative bacterial lipopolysaccharides: a human disease determinant? Infect Immun 76:454–465. doi:10.1128/IAI.00939-07. 18086818 PMC2223455

[55] Munford RS, Varley AW 2006 Shield as signal: lipopolysaccharides and the evolution of immunity to gram-negative bacteria. PLoS Pathog 2:e67. doi:10.1371/journal.ppat.0020067. 16846256 PMC1483240

[56] Bakaletz LO 2010 Immunopathogenesis of polymicrobial otitis media. J Leukoc Biol 87:213–222. doi:10.1189/jlb.0709518. 19843575 PMC2812561

[57] Man WH, Clerc M, de Steenhuijsen Piters WAA, van Houten MA, Chu M, Kool J, Keijser BJF, Sanders EAM, Bogaert D 2019 Loss of microbial topography between oral and nasopharyngeal microbiota and development of respiratory infections early in life. Am J Respir Crit Care Med 200:760–770. doi:10.1164/rccm.201810-1993OC. 30883192

[58] Biesbroek G, Tsivtsivadze E, Sanders EA, Montijn R, Veenhoven RH, Keijser BJ, Bogaert D 2014 Early respiratory microbiota composition determines bacterial succession patterns and respiratory health in children. Am J Respir Crit Care Med 190:1283–1292. doi:10.1164/rccm.201407-1240OC. 25329446

[59] Bosch A, Levin E, van Houten MA, Hasrat R, Kalkman G, Biesbroek G, de Steenhuijsen Piters WAA, de Groot PCM, Pernet P, Keijser BJF, Sanders EAM, Bogaert D 2016 Development of upper respiratory tract microbiota in infancy is affected by mode of delivery. EBioMedicine 9:336–345. doi:10.1016/j.ebiom.2016.05.031. 27333043 PMC4972531

[60] Wyllie AL, Chu MLJN, Schellens MHB, van Engelsdorp Gastelaars J, Jansen MD, van der Ende A, Bogaert D, Sanders EAM, Trzciński K 2014 *Streptococcus pneumoniae* in saliva of Dutch primary school children. PLoS One 9:e102045. doi:10.1371/journal.pone.0102045. 25013895 PMC4094488

[61] Bosch A, de Steenhuijsen Piters WAA, van Houten MA, Chu M, Biesbroek G, Kool J, Pernet P, de Groot PCM, Eijkemans MJC, Keijser BJF, Sanders EAM, Bogaert D 2017 Maturation of the infant respiratory microbiota, environmental drivers, and health consequences. a prospective cohort study. Am J Respir Crit Care Med 196:1582–1590. doi:10.1164/rccm.201703-0554OC. 28665684

[62] Davis NM, Proctor DM, Holmes SP, Relman DA, Callahan BJ 2018 Simple statistical identification and removal of contaminant sequences in marker-gene and metagenomics data. Microbiome 6:226. doi:10.1186/s40168-018-0605-2. 30558668 PMC6298009

[63] Oksanen J, Blanchet G, Friendly M, Kindt R, Legendre P, McGlinn D, Minchin P, O'Hara RB, Simpson G, Solymos P, Stevens MHH, Szoecs E, Wagner H 2019 vegan: community ecology package. R package version 2.5-6. https://CRAN.R-project.org/package=vegan.

[64] McMurdie PJ, Holmes S 2013 phyloseq: an R package for reproducible interactive analysis and graphics of microbiome census data. PLoS One 8:e61217. doi:10.1371/journal.pone.0061217. 23630581 PMC3632530

[65] Paulson JN, Stine OC, Bravo HC, Pop M 2013 Differential abundance analysis for microbial marker-gene surveys. Nat Methods 10:1200–1202. doi:10.1038/nmeth.2658. 24076764 PMC4010126

[66] Lahti L, Shetty S 2017 Tools for microbiome analysis in R. http://microbiome.github.com/microbiome.

[67] Wickham H 2016 ggplot2: elegant graphics for data analysis. Springer-Verlag, New York, NY.

[68] McHardy IH, Goudarzi M, Tong M, Ruegger PM, Schwager E, Weger JR, Graeber TG, Sonnenburg JL, Horvath S, Huttenhower C, McGovern DP, Fornace AJ Jr, Borneman J, Braun J 2013 Integrative analysis of the microbiome and metabolome of the human intestinal mucosal surface reveals exquisite inter-relationships. Microbiome 1:17. doi:10.1186/2049-2618-1-17. 24450808 PMC3971612

