# Supplementary material for: Niche- and Gender-Dependent Immune Reactions in Relation to the Microbiota Profile in Pediatric Patients with Otitis Media with Effusion
Source: Infect Immun. 2020 Sep 18;88(10):e00147-20. doi: 10.1128/IAI.00147-20 (PMC7504947; doi:10.1128/IAI.00147-20)
Supplement: Supplemental file 1 [file IAI.00147-20-s0001.pdf]

SUPPLEMENTAL TABLES

Supplemental Table 1. Patient characteristics and identified pathogen in the respective sample. [a] Age in months. [b] Pathogen identified by clinical microbiological analyses (culturing and/or 16S DNA. Abbreviations: RME, right middle ear effusion; LME, left middle ear effusion; NPH, nasopharynx sample; neg, negative; WR, without remark *i.e.,* no previous history of breached tympanic membrane; Myringot, previous myringotomy without ventilation tube insertion; VT, previous myringotomy with ventilation tube insertion; Perf AOM, documented history of perforated tympanic membrane due to acute otitis media; HI, *Haemophilus influenzae*; Spn, *Streptococcus pneumoniae*; Mrx, *Moraxella catarrhalis*; S.aur, *Staphylococcus aureus*; GAS, *Streptococcus pyogenes*; CoNS, coagulase negative staphylococci; A.otit, *Alloiococcus otitidis*; Staph, *Staphylococcus* spp and Neiss, *Neisseria* spp.

Patient characteristics			*RME*		*LME*		*NPH*
**Patient**	**Gender**	**Age[a]**	**Pathogen identified [b]**	**Comment**	**Pathogen identified [b]**	**Comment**	**Pathogen identified [b]**
**Pat 2**	Male	110	neg	WR	neg	WR	neg
**Pat 3**	Male	75	HI	WR	neg	WR	Spn. Mrx. HI
**Pat 4**	Male	51	neg	WR	neg	WR	Mrx
**Pat 6**	Male	52	neg	WR	neg	WR	Mrx
**Pat 8**	Female	43	neg	Myringot.	neg	WR	Mrx
**Pat 9**	Male	92	neg	VT	neg	V.T.	neg
**Pat 10**	Male	60	neg	WR	neg	WR	Mrx
**Pat 12**	Female	71	neg	WR	neg	WR	HI
**Pat 13**	Female	74	S.aur. Mrx	WR	A. otit	WR	S.aur
**Pat 14**	Female	57	HI spec	WR	neg	WR	GAS. Mrx
**Pat 15**	Male	48	GAS, CoNS	Myringot.	neg	Myringot.	GAS. Mrx
**Pat 16**	Female	58	A.otit	WR	A.otit	WR	Mrx
**Pat 17**	Female	72	neg	WR	A.otit	WR	Mrx
**Pat 19**	Female	61	neg	WR	neg	WR	Mrx
**Pat 20**	Female	86	neg	VT	Staph. CoNS	VT	HI. Mrx
**Pat 21**	Female	24	HI. CoNS	WR	CoNS	WR	HI. GAS
**Pat 22**	Female	59	HI	WR	neg	WR	Mrx. HI
**Pat 25**	Male	103	neg	VT	neg	VT	Mrx
**Pat 27**	Male	68	neg	VT	neg	VT	Mrx
**Pat 28**	Female	38	neg	WR	neg	WR	Spn. HI
**Pat 29**	Male	47	HI	VT	neg	VT	HI. Mrx
**Pat 30**	Male	74	S.aur	WR	S.aur	WR	neg
**Pat 31**	Female	35	neg	WR	neg	WR	Spn. Mrx
**Pat 33**	Male	42	neg	WR	neg	WR	Spn. Mrx
**Pat 34**	Female	46	HI. S.aur	WR	HI	WR	HI
**Pat 35**	Male	32	neg	Perf AOM	neg	WR	HI. Neiss
**Pat 37**	Male	44	neg	WR	neg	WR	Mrx

Supplemental Table 2. Correlation coefficient of nMDS plot from Figure 1B, with *P*- and q-values indicated.

Comparison	***R2***	***P*-value**	***q*-value**
EEC vs MEE	0.0442	0.0619	0.0619
EEC vs NPH	0.2515	**0.0010*****	0.0015
MEE vs NPH	0.2163	**0.0010*****	0.0015

Supplemental Table 3. Leukocyte populations and inflammatory mediators in MEEs and NPH samples. [a] Values are median percentage ±SEM (*n*) of leukocyte subpopulation of all viable cells. [b] Exact *P*-value by Mann-Whitney Wilcoxon test. [c] percentage (%) of naïve or memory cells of all $CD4^+$ or $CD8^+$ T lymphocytes as determined by flow cytometry. [d] [% $CD4^+$ cells of all viable cells / % $CD8^+$ cells of all viable cells]. [e] Median concentration of indicated mediator in pg/mL ±SEM (*n*), as assessed by cytometric cytokine bead array (CBA).

Leukocyte subpopulation[a]	***MEE***	***NPH***	***P*-value**[b]
$CD66b^+$ neutrophils	49.80 ± 5.84 (28)	16.76 ± 6.35 (17)	0.110
$CD14^+HLA\text{-}DR^+$ monocytes/macrophages	1.31 ± 0.55 (28)	1.17 ± 1.35 (17)	0.890
$CD3^+$ T lymphocytes	4.65 ± 3.61 (28)	15.00 ± 2.88 (17)	0.185
$CD4^+$ T-helper cells	5.18 ± 1.51 (20)	6.71 ± 1.72 (14)	0.568
% $CD45RA^+$ naïve $CD4^+$ cells [c]	3.84 ± 8.21 (12)	4.95 ± 1.2 (9)	0.638
% $CD45RO^+$ memory $CD4^+$ cells [c]	87.35 ± 8.82 (12)	89.10 ± 9.73 (9)	0.931
$CD8^+$ cytotoxic T cells	1.76 ± 0.83 (20)	6.35 ± 2.15 (15)	**0.002****
% $CD45RA^+$ naïve $CD8^+$ cells [c]	26.45 ± 5.66 (12)	8.07 ± 2.03 (10)	**0.014***
% $CD45RO^+$ memory $CD8^+$ cells [c]	63.00 ± 6.36 (13)	75.45 ± 5.66 (10)	0.455
CD4/CD8 T cell ratio [d]	2.52 ± 0.54 (20)	0.86 ± 0.76 (15)	0.542
$CD19^+$ B lymphocytes	0.98 ± 0.66 (28)	23.90 ± 6.57 (17)	**<0.0001*****
$CD56^+CD3^-$ NK cells	0.065 ± 0.37 (28)	0.00 ± 0.07 (16)	0.244
$CD56^+CD3^+$ NKT cells	0.00 ± 0.06 (27)	0.00 ± 0.03 (17)	>0.999
Inflammatory mediator[e]	***MEE***	***NPH***	***P*-value**[b]
IL-6	155.43 ± 348.36 (50)	44.83 ± 29.93 (27)	0.063
IL-8	1963.34 ± 603.07 (50)	2141.41 ± 677.84 (27)	0.666
IL-10	2.95 ± 4.29 (50)	2.46 ± 2.31 (27)	0.549
TNF	0.73 ± 4.32 (50)	3.61 ± 1.89 (27)	**0.013***
IL-1β	9.43 ± 68.49 (50)	94.90 ± 43.09 (27)	**0.002****

Supplemental Table 4. Leukocyte populations and inflammatory mediators in MEEs and NPH samples. Samples were stratified according to age at myringotomy (cut-off median age of patients; 58 months). [a] Values are median percentage ±SEM (*n*) of leukocyte subpopulation of all viable cells. [b] Exact *P*-value by Mann-Whitney Wilcoxon test. [c] percentage (%) of naïve or memory cells of all $CD4^{+}$ or $CD8^{+}$ T lymphocytes as determined by flow cytometry. [d] [% $CD4^{+}$ cells of all viable cells / % $CD8^{+}$ cells of all viable cells]. [e] Median concentration of indicated mediator in pg/mL ±SEM (*n*), as assessed by cytometric cytokine bead array (CBA).

	MEE			***NPH***		
Leukocyte subpopulation[a]	***<58mo***	***>58mo***	***P-value***[b]	***<58mo***	***>58mo***	***P-value***[b]
$CD66b^{+}$ neutrophils	75.1 ± 9.42 (13)	44.69 ± 6.16 (15)	**0.022***	32.56 ± 9.58 (8)	5.96 ± 8.24 (9)	0.277
$CD14^{+}HLA\text{-}DR^{+}$ monocytes/macrophages	2.61 ± 0.81 (13)	0.6 ± 0.78 (15)	0.768	2.08 ± 1.34 (8)	0 ± 2.35 (9)	0.162
$CD3^{+}$ T lymphocytes	2.77 ± 2.09 (13)	4.96 ± 6.06 (15)	0.085	13.9 ± 2.52 (8)	25.9 ± 4.72 (9)	0.144
$CD4^{+}$ T-helper cells	5.18 ± 1.39 (6)	4.99 ± 2.08 (14)	>0.999	8.75 ±2.57 (6)	5.30 ± 2.43 (8)	0.573
% $CD45RA^{+}$ naïve $CD4^{+}$ cells [c]	2.33 ± 13.73 (5)	10.8 ± 10.86 (7)	0.399	9.76 ± 1.82 (5)	4.65 ± 1.11 (4)	0.286
% $CD45RO^{+}$ memory $CD4^{+}$ cells [c]	94.2 ± 14.37 (5)	75.6 ± 11.41 (7)	0.213	88.2 ± 2.79 (5)	89.4 ± 21.77 (4)	0.905
$CD8^{+}$ cytotoxic T cells	1.58 ± 0.19 (6)	1.96 ± 1.15 (14)	0.534	6.35 ± 2.93 (7)	6.90 ± 3.28 (8)	0.955
% $CD45RA^{+}$ naïve $CD8^{+}$ cells [c]	8.33 ± 9.78 (5)	33.3 ± 5.61 (7)	0.073	8.62 ± 1.55 (5)	7.14 ± 4.01 (5)	0.691
% $CD45RO^{+}$ memory $CD8^{+}$ cells [c]	75.0 ± 8.65 (5)	59.4 ± 8.65 (8)	0.210	75.0 ± 9.34 (5)	83.0 ± 5.85 (5)	0.222
CD4/CD8 T cell ratio [d]	3.4 ± 0.64 (6)	2.19 ± 0.74 (13)	0.124	1.34 ± 1.12 (7)	0.86 ± 1.21 (7)	0.463
$CD19^{+}$ B lymphocytes	1.08 ± 0.57 (13)	0.3 ± 1.13 (15)	0.708	25.7 ± 10.04 (8)	21.6 ± 9.2 (9)	0.838
$CD56^{+}CD3^{-}$ NK cells	0.0 ± 0.17 (13)	0.56 ± 0.64 (15)	0.289	0.0 ± 0.06 (7)	0.18 ± 0.11 (9)	0.306
$CD56^{+}CD3^{+}$ NKT cells	0.0 ± 0.0 (12)	0.0 ± 0.10 (15)	0.226	0.0 ± 0.07 (8)	0.0 ± 0.0 (9)	0.206
Inflammatory mediator[e]	***<58mo***	***>58mo***	***P-value***[b]	***<58mo***	***>58mo***	***P-value***[b]
IL-6	103.6 ± 589.9 (28)	105.9 ± 159.6 (26)	0.602	44.6 ± 20.5 (14)	44.8 ± 58.9 (13)	0.905
IL-8	2272.2 ± 959.3 (28)	1149.7 ± 526.5 (26)	0.265	1883.6 ± 478.2 (14)	2344.9 ± 1320.3 (13)	0.607
IL-10	2.9 ± 6.5 (28)	2.3 ± 4.5 (26)	0.721	2.1 ± 1.8 (14)	2.5 ± 4.4 (13)	0.838
TNF	0.7 ± 7.6 (28)	0.2 ± 1.2 (26)	0.340	3.7 ± 3.2 (14)	3.2 ± 1.9 (13)	0.693
IL-1β	4.9 ± 115.3 (28)	8.5 ± 40.3 (26)	0.515	108.8 ± 60.3 (14)	76.8 ± 63.3 (13)	0.519

Supplemental Table 5. Spearman correlation of inflammatory cells and mediators in NPH samples. The potential correlation between inflammatory cells and mediators in NPH samples was assessed by Spearman r correlation. R values and significance is indicated. * $P < 0.05$. Abbreviations; Neu, neutrophils ($CD66b^+$); Mo-Macr, monocytes/macrophages ($CD14^+HLA\text{-}DR^+$); T cells ($CD3^+$); $CD8^+$ cytotoxic T cells; $CD4^+$ T-helper cells; CD4/CD8, %$CD4^+$ cells / % $CD8^+$ cells; B cells ($CD19^+$); NK cells ($CD56^+CD3^-$) and NKT cells ($CD56^+CD3^+$).

	Leukocyte population								
Mediator	*Neu*	*Mo-Macr*	*T cells*	*CD8 Tc*	*CD4 Th*	*CD4/CD8*	*Bc*	*NK*	*NKT*
IL-8	0.284	0.210	0.105	-0.248	0.179	-0.314	-0.179	0.149	0.053
IL-1β	0.135	0.134	0.006	0.108	-0.207	0.068	0.107	0.035	**0.486***
IL-6	0.032	-0.125	0.278	0.090	0.089	-0.103	0.153	-0.041	-0.053
IL-10	-0.055	-0.014	0.084	-0.097	-0.170	-0.150	0.086	-0.011	-0.272
TNFα	0.248	-0.147	0.177	-0.152	0.089	-0.262	-0.071	-0.093	0.280
IL10/TNF	-0.121	0.276	0.013	-0.181	-0.046	-0.170	0.075	0.265	-0.401
IL-1ra	-0.262	0.029	0.332	-0.033	-0.060	-0.253	-0.007	0.209	-0.103
IL-2	0.064	-0.007	0.095	0.014	0.014	-0.185	-0.113	-0.137	0.381
IL-4	-0.009	-0.079	0.150	0.146	-0.061	-0.091	-0.013	-0.026	0.310
IL-5	0.012	-0.241	0.156	0.302	0.067	0.123	0.006	0.258	0.480
IL-7	-0.242	0.014	0.260	0.151	-0.008	-0.132	0.108	0.220	0.276
IL-9	0.015	0.452	-0.024	-0.264	0.082	-0.368	-0.011	-0.030	0.172
IL-12	0.084	0.014	0.135	0.006	0.299	-0.246	-0.042	-0.107	0.381
IL-13	-0.164	0.102	0.284	0.028	0.163	-0.213	0.049	0.268	0.208
IL-15	0.215	-0.155	0.069	0.208	0.054	0.101	-0.110	0.029	0.474
IL-17	0.196	0.155	-0.046	0.038	-0.104	-0.104	-0.095	-0.134	0.310
Eotaxin	-0.037	-0.088	0.345	0.236	-0.115	0.077	0.121	0.448	-0.241
FGF basic	0.068	0.065	0.112	0.440	-0.297	0.473	0.398	0.149	0.378
G-CSF	-0.253	0.209	0.297	-0.011	0.203	-0.253	0.051	0.134	0.310
GM-CSF	-0.123	-0.057	0.123	0.311	-0.183	0.128	0.234	0.209	0.384
IFNγ	-0.284	-0.038	0.327	-0.214	0.011	-0.390	-0.020	0.078	0.034
IP-10	0.083	0.329	0.196	-0.100	0.078	-0.228	-0.002	0.577	-0.106
MCP-1	0.187	0.025	0.130	-0.071	0.071	-0.264	-0.156	0.123	0.172
MIP-1α	0.244	0.178	0.064	-0.220	0.269	-0.473	-0.288	-0.131	-0.103
PDGF-bb	0.071	0.262	-0.281	0.147	-0.050	0.047	-0.129	-0.111	0.420
MIP-1β	0.393	0.187	0.055	-0.192	0.242	-0.363	-0.222	-0.123	-0.172
RANTES	-0.108	0.299	0.099	-0.181	0.071	-0.302	0.077	0.011	0.310
VEGF	0.187	0.107	0.147	0.011	0.072	-0.138	-0.111	0.103	0.243
M-CSF	0.219	0.401	-0.100	-0.074	-0.130	-0.091	-0.069	-0.040	0.173
IL-18	0.125	-0.250	0.191	0.489	-0.401	0.588	0.231	0.198	0.172

Supplemental Table 6. PermANOVA analysis of β-diversity and clinical parameters.

All samples				
Parameter	***Number of samples***	***Degrees of freedom***	***R2***	***P*-value**
Niche (NPH, MEE, EEC)	74	2	0.225	**0.001**[***]
Age (<58 mo, >58 mo)	74	1	0.019	0.110
Gender (male, female)	74	1	0.009	0.507
Affected ears (uni-, bilateral)	74	1	0.012	0.342
Inflammation type (neutrophil / lymphocyte predominance)	26	2	0.048	0.267
Middle ear effusion samples				
Parameter	***Number of samples***	***Degrees of freedom***	***R2***	***P*-value**
Age (<58 mo, >58 mo)	24	1	0.065	0.220
Gender (male, female)	24	1	0.042	0.386
Affected ears (uni-, bilateral)	24	1	0.067	0.176
Nasopharyngeal samples				
Parameter	***Number of samples***	***Degrees of freedom***	***R2***	***P*-value**
Age (<58 mo, >58 mo)	20	1	0.025	0.870
Gender (male, female)	20	1	0.053	0.422
Affected ears (uni-, bilateral)	20	1	0.054	0.420

SUPPLEMENTAL FIGURES

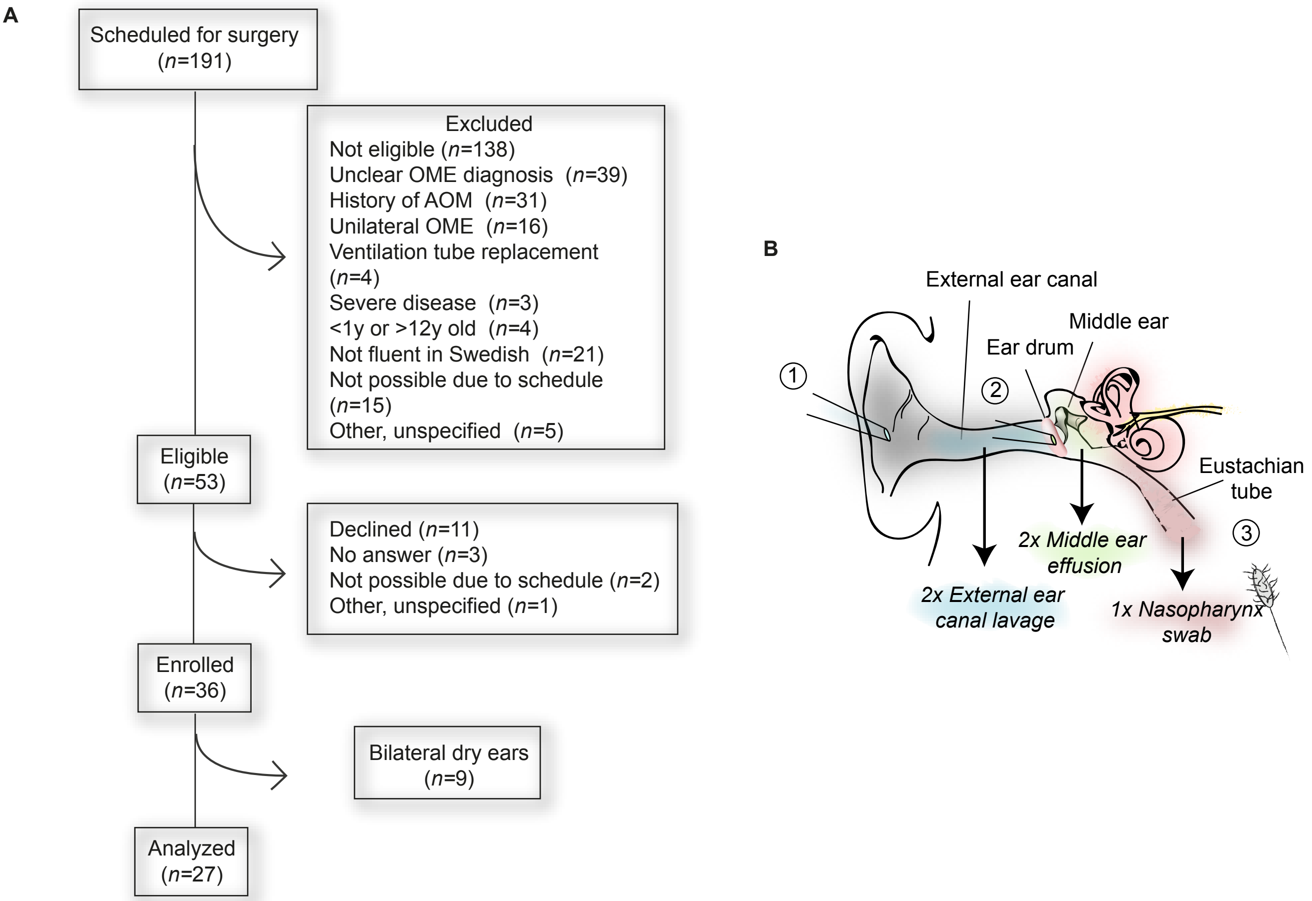

Supplemental Figure 1. Overview of the patient inclusion and sample collection. A. A schematic summary of the patient inclusion. Of 191 patients scheduled for surgery, 36 patients were enrolled in the study. Nine patients were excluded due to bilateral dry ears. The remaining 27 patients were analyzed for inflammatory parameters and microbiota composition. **B.** Sample collection was performed as follows: 1) The external ear canals of the enrolled patients were first washed with sterile saline for analyses of the external ear canal (EEC) microbiota composition. 2) After a subsequent wash with 70% denatured alcohol. the middle ear effusions (MEEs) were collected and analyzed for inflammatory cells and mediators as well as microbiota composition. 3) Nasopharyngeal (NPH) swabs from all patients were reconstituted in sterile saline and analyzed for inflammatory cells and mediators as well as microbiota composition.

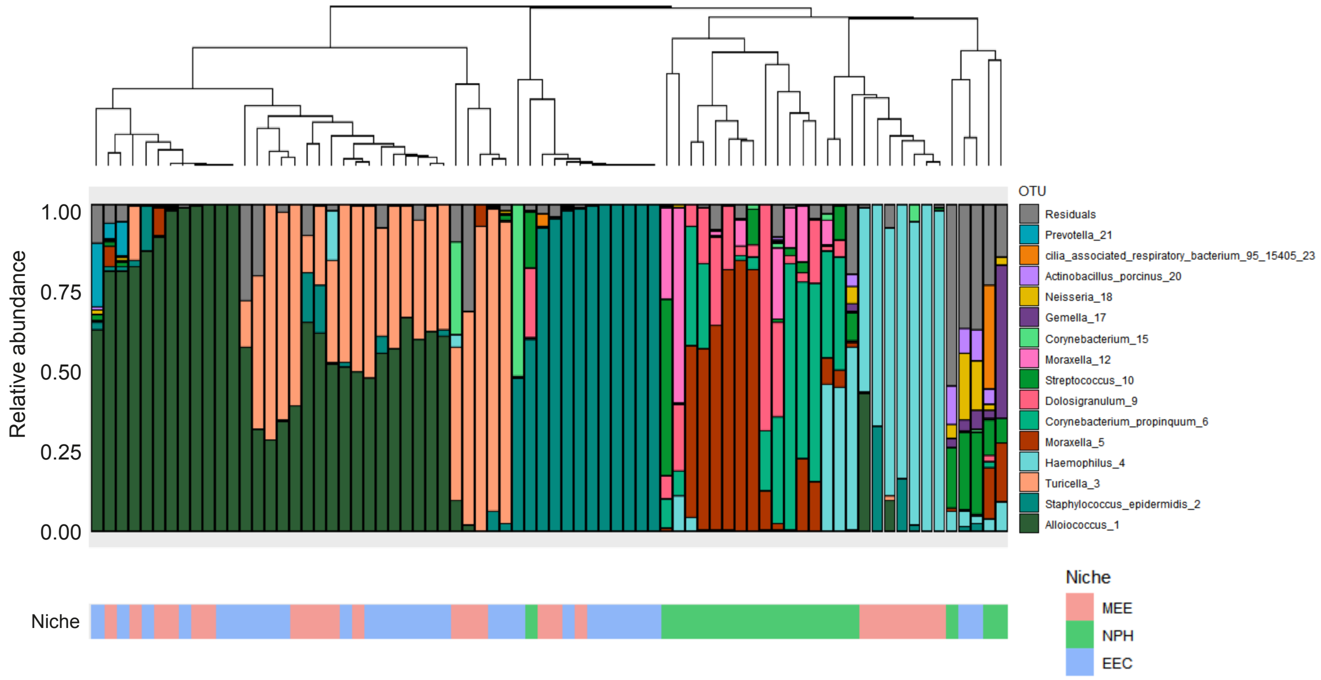

Supplemental Figure 2. Niche-based hierarchical clustering of microbiota profiles. Dendrogram visualizing an average linkage hierarchical clustering of all study samples on the basis of the Bray–Curtis dissimilarity matrix. Stacked bar charts show the relative abundance of the 15 highest-ranked operational taxonomic units (OTUs) and of residual bacteria. Niche identity is depicted adjacent to the branch ends. NPH samples mainly clusters separately and are dominated by *Moraxella* (5), *Corynebacterium propinquum* (6), *Streptococcus* (10), *Haemophilus* (4) and *Dolosigranulum* (9). MEEs and EEC lavages mainly clusters together, although MEEs also shared some features with NPH samples with a high relative abundance of *Alloiococcus* (1), followed by *Haemophilus* (4), *Turicella* (3) and *Staphylococcus epidermidis* (2), whereas EEC lavages displayed high relative abundance of *Alloiococcus* (1), *S. epidermidis* (2) and *Turicella* (3).

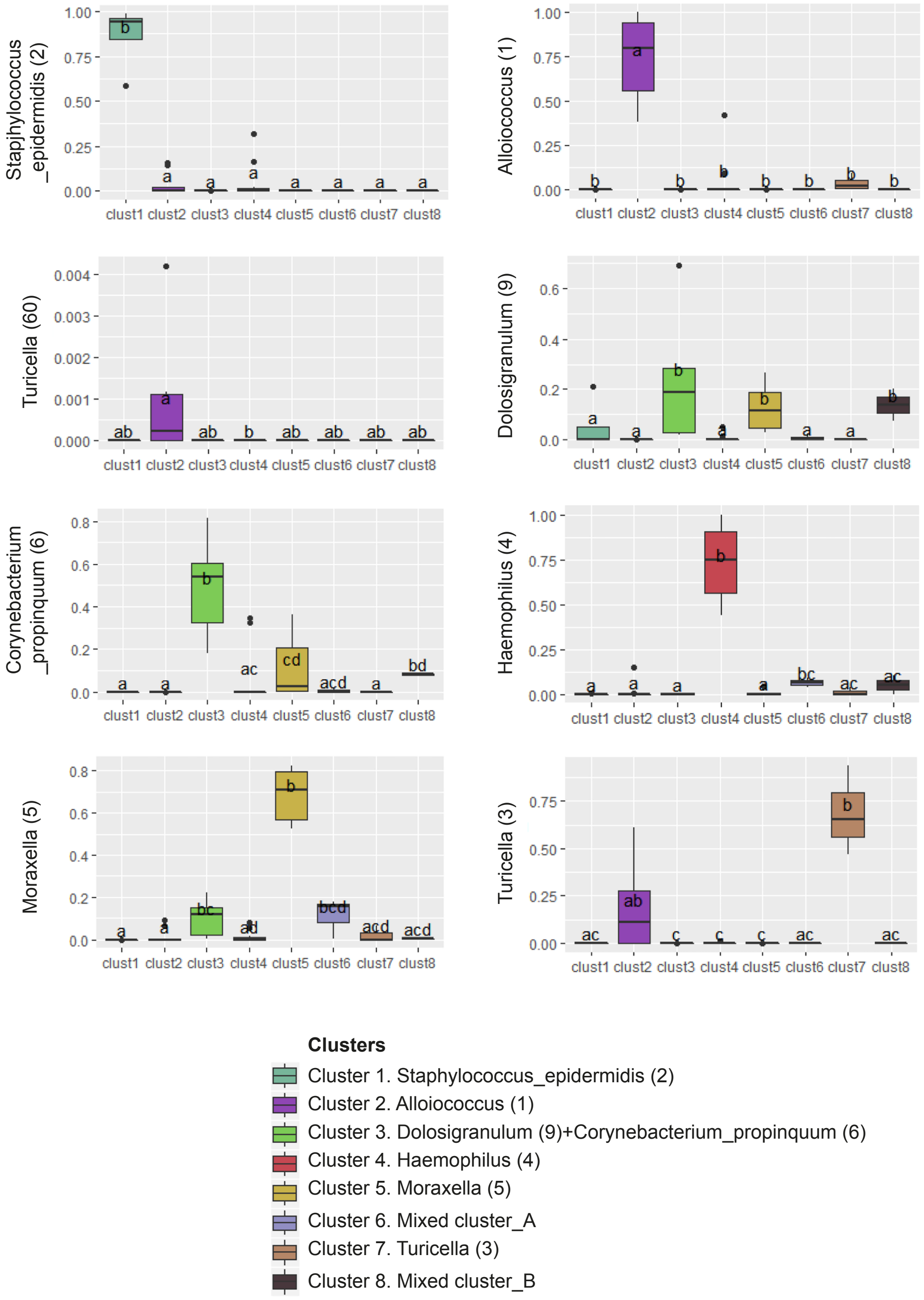

Supplemental Figure 3. Biomarker species stratified according to the identified clusters. Hierarchical clustering and random forest analysis identified eight clusters and biomarker species. Boxplot showing relative abundance of the identified biomarker species, stratified by microbiome clusters. Multivariable linear regression modeling was used to assess the association between the log-transformed biomarker abundance and the clusters corrected by niche with Tukey post hoc test, values with unlike letters differed significantly.

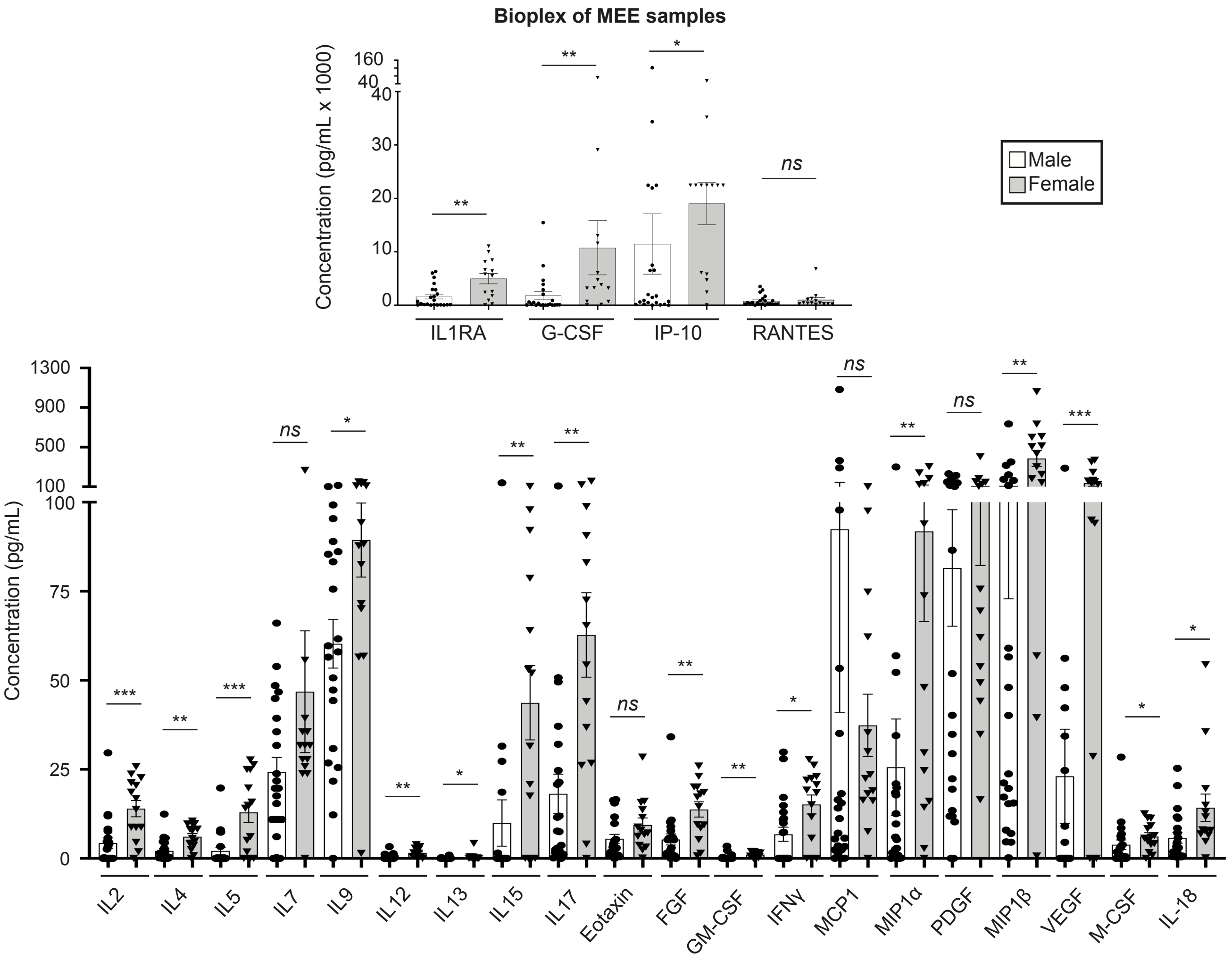

Supplemental Figure 4. Bio-plex analyses of MEE samples. MEE samples were analyzed by Bio-plex multiple cytokine assay for inflammatory mediators and stratified according to gender. Concentration (pg/mL) of indicated inflammatory mediators in individual samples with bars representing mean concentration +/- SEM shown. Male MEEs (black circles, white bars) *n*=22 and female MEEs (black triangles, grey bars) *n*=14. All statistics by Mann Whitney U test * $P < 0.05$, ** $P < 0.01$ and *** $P < 0.001$.
